# Synthetic microbiomes in bioengineered rhizospheres: new frontiers for climate-resilient agriculture

**DOI:** 10.3389/fmicb.2026.1780132

**Published:** 2026-04-09

**Authors:** Krishnendu Adhikary, Samy Selim, Riya Sarkar, Krishnendu Ganguly, Joy Das, Mohammed S. Almuhayawi, Mohammed H. Alruhaili, Hattan S. Gattan, Prithviraj Karak

**Affiliations:** 1 Department of Medical Laboratory Technology, Paramedical College Durgapur, Durgapur, West Bengal, India; 2 Department of Clinical Laboratory Sciences, College of Applied Medical Sciences, Jouf University, Sakaka, Saudi Arabia; 3 Department of Medical Laboratory Technology, Dr. B. C. Academy of Professional Courses, Fuljhore, Durgapur, West Bengal, India; 4 School of Pharmaceutical Sciences, Lovely Professional University, Phagwara, Punjab, India; 5 Department of Clinical Microbiology and Immunology, Faculty of Medicine, King Abdulaziz University, Jeddah, Saudi Arabia; 6 Special Infectious Agents Unit, King Fahad Medical Research Center, King Abdulaziz University, Jeddah, Saudi Arabia; 7 Department of Medical Laboratory Sciences, Faculty of Applied Medical Sciences, King Abdulaziz University, Jeddah, Saudi Arabia; 8 Department of Physiology, Bankura Christian College, Bankura, West Bengal, India

**Keywords:** climate change, microbial genomics, rhizospheres, sustainable agriculture, synthetic microbiomes

## Abstract

Climate change poses significant threats to global agricultural productivity, necessitating innovative strategies to ensure food security and ecological sustainability. One promising avenue lies in the deliberate design and deployment of synthetic microbiomes and engineered rhizospheres to enhance plant resilience under environmental stress. This review places particular emphasis on multi-kingdom microbial interactions including bacteria, fungi, protists, and archaea and their potential for tailored, stress-specific applications within engineered rhizosphere systems. By integrating knowledge from microbial ecology, genomics, and systems biology, researchers have begun to unravel the complex interactions between plants and their associated microbial communities. Engineered microbial assemblies tailored to specific host plants and environmental conditions have shown potential in stabilizing crop performance during drought, salinity, and nutrient limitations. Moreover, the manipulation of root exudation patterns and soil physicochemical properties can be harnessed to recruit beneficial microbes and suppress harmful ones. The review also examines the role of synthetic biology tools, such as CRISPR-based genome editing and metabolic pathway engineering, in optimizing microbial traits for enhanced plant support. However, knowledge gaps remain in understanding multi-kingdom dynamics, optimizing SynComs for specific environmental contexts, and translating laboratory successes to reliable, field-scale applications. Additionally, advances in high-throughput screening, machine learning, and metagenomic profiling are accelerating the identification of key microbial taxa and functions relevant to plant health. Despite these promising developments, challenges remain in scaling these approaches for field applications and ensuring their ecological safety and consistency. This review explores the need for interdisciplinary efforts to translate laboratory insights into field-ready technologies, ultimately contributing to the development of climate-resilient and sustainable agricultural systems.

## Graphical Abstract

**Figure:**
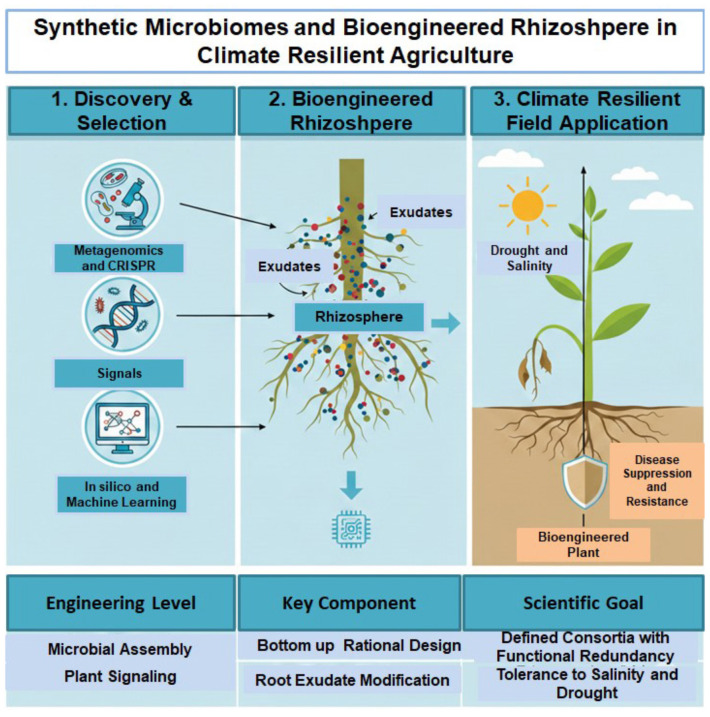
Synthetic microbiomes and bioengineered rhizospheres in climate-resilient agriculture.

## 1 Introduction

A microbes diverse community such as archaea, bacteria, fungi, and protists which coexists with plants and is collectively denoted as plant microbiota and along with their habitats, genomes, and the surrounding environmental factors together it comprehends as plant microbiome, which is presently considered an comprehensive plant trait with functional capabilities that contribute to plant host nutrition, development and immunity (Teixeira et al., 2019). Nowadays, as a modern tool for promoting sustainable agriculture, biofertilizers based on plant growth–promoting rhizobacteria (PGPR) are gaining increasing attention due to their ability to enhance root nutrient acquisition. However, the application of traditional PGPR inoculants to enhance the acquisition of nutrients by plants and their growth promotion is affected by inconsistencies caused by poor establishment, lower persistence of the inoculants, and poor competitor overlap for the indigenous micro-organisms in the soil, especially under stress conditions resulting from climate change (Hassani et al., 2018; Compant et al., 2021; Yang et al., 2023). The application of traditional inoculants composed of a few members of micro-organism populations does not take into consideration the functionality of redundancy and the complementarity of the natural populations of micro-organisms (Jacoby et al., 2021). In contrast, synthetic microbial communities that are composed of a combination of different micro-organism populations possess ecological reliability, reproducibility, and robustness by considering the cooperative effects that encompass different bacterial and fungal populations. Such a tool is reliable for overcoming the inconsistencies facing traditional PGPR inoculants (Pascale et al., 2020). As promising strategies to foster synergistic interactions the various microbiome engineering methods that involve microbial communities manipulation have emerged, eventually enhancing the overall health and growth of the host plant. These approaches comprise the creation of synthetic communities (SynComs) and the host-mediated microbiome engineering (HMME) (Teixeira et al., 2019; Yang et al., 2023). The benefits of these approaches are improving nutrient availability in the rhizosphere (with the consequent decrease in chemical fertilizer supplementation with the related environmental benefits) also shows positive effects of the rhizosphere microbiome to the plant health, by increasing the capability of plant to cope with biotic and abiotic stresses (Yang et al., 2023). In specific plant compartments the plants gradually enrich microbes by creating microbial habitats which typically start from the bulk soil and can move into above-ground internal plant tissues. Hence, the plant microbiome composition is compartment specific and is divided into rhizosphere, endosphere, and phyllosphere (Compant et al., 2021). The healthy and asymptomatic plants maintain a complex relationship with rhizosphere microbiota, which supports plant performances (Hassani et al., 2018). By altering soil pH, oxygen availability soil structure, and by providing carbon-rich energy source, the plants influence the composition and activity of their rhizosphere microbiome (Jacoby et al., 2021). Plant root exudates comprise a chemically diverse mixture of primary and secondary metabolites, many of which exhibit bioactive properties that affect microbial composition and the surrounding environment (Pascale et al., 2020). By the release of signaling molecules the microbes and plants are capable of influencing their plant host however rhizosphere-associated micro-organisms perceive and interpret signals they themselves produce. The main consequences of this communication are related to the induction of plant immunity, stress tolerance, overall growth, health, nutrition, and the maintenance of associated rhizosphere microbiomes. Some of the examples for signaling molecules are diffusible signal factors, N/acyl homoserine lactones (AHLAs), phytohormone-like molecules, diketopiperazines, and volatile organic compounds (Bailly and Weisskopf, 2012; Granato et al., 2019). By 2050, crop yields in many regions could decline by up to 30% due to climate change-related heat stress, drought, and water scarcity threatening global food security. Temperature changes, drought, and changes in soil chemistry associated with climate changes impose stress factors on crop productivity (Neu et al., 2021; Kuźniar et al., 2023; Ma H. et al., 2025). However, the functionality of the rhizosphere is also affected due to these changes. Though the impact of changes in composition due to microbiomes in the rhizosphere with different environmental conditions has been established as well as the effects of changes in the microbiome in relation to stress conditions, the majority of the literature is not based on cause-and-effect relationships or a mechanistic model, especially for the interactions of fungi and other microbiomes. In this regard, the lack of well-controlled reproducible methods to address the issue is a solution to the problem due to climate changes (Granato et al., 2019). Bacteria have developed a variety of strategies that enable them to thrive and successfully compete in a variety of ecological settings. It significantly affects the host organism and modifies the rhizosphere’s microbial makeup. Such interactions include exploitative competition, where microbes compete for limited resources and the more efficient ones restrict access for others, and interference competition, where microbes produce various antibacterial or antimicrobial compounds to inhibit their rivals. While foundational studies have greatly expanded our understanding of mycobiome composition and ecological roles, the inherent complexity and variability of natural fungal communities limit our ability to establish causal relationships (Kuźniar et al., 2023; Wang et al., 2024; Taerum et al., 2025). Environmental heterogeneity, host specificity, and multi-kingdom interactions further obscure functional mechanisms. To overcome these constraints, researchers increasingly employ synthetic fungal communities defined, reproducible assemblages of selected taxa that enable systematic dissection of mycobiome-driven processes under controlled conditions (Negre Rodríguez et al., 2025). Through the production of small diffusible molecules the interference competition can be achieved which functions among physically separated bacteria or relies on lethal effector proteins secretion to antagonize competing microbes. Our aim was to develop innovative strategies for enhancing plant health and resilience, understanding how rhizosphere communities interact provides valuable insights can be used (Neu et al., 2021; Ma H. et al., 2025). Despite rapid advances in mycobiome research, our understanding of how individual fungal taxa and their interactions drive host and ecosystem-level functions remains limited (Lei et al., 2025). Most existing studies rely on descriptive, correlative analyses of complex natural communities, which hampers the ability to establish causality, reproducibility, and mechanistic insight. In particular, there is a lack of integrative frameworks that connect foundational mycobiome knowledge with experimental approaches capable of disentangling fungal–fungal and fungal–host interactions (Negre Rodríguez et al., 2025; Taerum et al., 2025). Consequently, the potential of synthetic fungal communities as tractable model systems has not been systematically synthesized or critically evaluated (Negre Rodríguez et al., 2025). A central challenge in mycobiome research is the inability to move beyond descriptive characterization toward a mechanistic understanding of how fungal communities influence host and ecosystem functions. This review addresses this gap by consolidating current mycobiome research and examining how synthetic communities can be strategically designed and applied to advance mechanistic understanding (Lei et al., 2025). In this review, we have emphasized on synthetic microbiomes and bioengineered rhizospheres represented a science-guided, ecologically informed, and climate-ready strategy to stabilize crop productivity, restore soil function, and mitigate climate change; this is how laboratory innovation translates into a sustainable field application. Although high-throughput sequencing and community profiling have revealed extensive fungal diversity, these approaches are largely correlative and provide limited insight into causal relationships, functional redundancy, or context-dependent interactions (Xu et al., 2025). Moreover, natural mycobiomes are shaped by environmental heterogeneity, host genotype, and multi-kingdom interactions, making experimental reproducibility and hypothesis testing difficult. As a result, current methodologies are insufficient to disentangle the specific contributions of individual fungal taxa and their interactions. This review addresses this critical gap by examining how synthetic fungal communities can serve as controlled, reductionist systems to overcome these limitations and enable mechanistic interrogation of mycobiome function (Singh et al., 2025). Early efforts in microbiome engineering emerged from single-strain inoculation and probiotic approaches, which sought to manipulate host-associated microbiota through the introduction of individual microbial taxa. As the limitations of these reductionist strategies became apparent, the field evolved toward designing multi-member synthetic communities to better capture microbial interactions while retaining experimental control (Pradhan et al., 2025).

## 2 Natural rhizosphere microbiome: composition and functions

### 2.1 Core microbiome concept (bacteria, fungi, archaea, protists)

The idea of a core microbiome in the rhizosphere has transformed an initial taxonomic checklist approach into a cross-kingdom, function-focused model that acknowledges both stable and dynamic components that are involved in plant health and resilience. Traditionally defined as taxa consistently associated with a host across space and time, the core now encompasses recurrent functional traits (e.g., nitrogen transformation, phosphorus solubilization, hormone modulation, disease suppression) that may be encoded by taxonomically variable members, in other words, a functional core can persist even when identities shift (Neu et al., 2021; Ma H. et al., 2025). Recent multi-omics and network research reveals that bacteria (usually Proteobacteria, Actinobacteria, Firmicutes, and Bacteroidetes) constitute the most numerous and metabolically flexible part of the core, promoting nutrient exchange and promoting the growth of plants, whereas fungi, from mutualistic arbuscular mycorrhizae to saprotrophic and endophytic groups of taxa, complement each other in organic matter turnover, pathogen defense, and abiotic stress countering (Kuźniar et al., 2023; Ma H. et al., 2025). Archaea, less abundant, with repeated occurrence in core assemblies with particular functions in nitrogen and carbon cycling in extreme or oligotrophic environments, point to their disproportionate functional significance despite taxonomic scarcity (Ma H. et al., 2025). More importantly, protists are top-down regulators, which can regulate the bacterial composition and functional outputs via selective grazing and trophic interactions to influence emergent core functions and network stability (Wang et al., 2024; Taerum et al., 2025). Recent efforts also emphasize that the core is context-specific: a combination of plant genotype, soil type, and climate conditions selectively retains as core elements which taxa and functions, leading to core microbiomes locally adapted but consisting of conserved functional modules, a fact that guides the rational design of synthetic communities (SynComs) and microbiome engineering to climate-resilient agriculture (Lei et al., 2025; Negre Rodríguez et al., 2025). Methodologically, integrated amplicon surveys, shotgun metagenomics, metatranscriptomics, and manipulative SynCom experiments are now used to define cores to make the distinction between resident, active, and causal members and transient passengers, a necessary refinement in translating core concepts into high-impact and field-relevant bioengineering strategies (Neu et al., 2021; Xu et al., 2025).

#### 2.1.1 Key functional roles: nutrient mobilization, growth promotion, disease suppression

The rhizosphere is a dynamic biochemical hot spot, which entails coordination of nutrient mobilization, growth promotion, and multi-layered disease manipulation processes by microbial consortia, which collectively underlies the foundations of plant productivity and resistance to climatic stress. Biological releases of nutrients involve biologically mediated fixations of nitrogen (symbiotic fixation and free-living fixation), mineral weathering, and mineralization of organic matter; enzymatic solubility of phosphorus; and microbial fixation of siderophores and chelators that increase the contribution of nutrients by Fe, Zn, and other micronutrients (Table 1). All processes contribute to reducing overreliance of plants on artificial fertilizers and alleviating nutrient shortages in unpredictable soils (Pradhan et al., 2025; Singh et al., 2025). Other growth-stimulating mechanisms include microbial synthesis of phytohormones (auxins, cytokinins, and gibberellins); volatile organic compounds synthesized that modify root architecture and water-stress signaling; enzymatic activities like ACC deaminase, which suppresses ethylene-induced growth arrest under abiotic stress; and processes that increase root surface area, water foraging, and nutrient uptake efficiency (Chen Q. et al., 2024; Gu et al., 2025). Suppression of diseases occurs through direct antagonism (antibiotics, lytic enzymes, bacteriocins), competitive displacement of pathogens, both niches and resources, induction of plant systemic defenses (ISR), and trophic interactions, especially protist grazing and bacteriophage interactions, which convert microbial assemblages to disease-suppressive networks *in situ* (Ping et al., 2024; Amutuhaire et al., 2025). Recent multi-omics and manipulation experiments highlight that these functions are often distributed across taxa (functional redundancy and cross-kingdom complementarity) and are highly context-dependent, shaped by root exudation patterns, soil physicochemistry and climate; this ecological nuance both complicates and empowers the rational design of synthetic communities and bioengineered rhizospheres aimed at delivering consistent nutrient- and pathogen-management services under field conditions (Alzate Zuluaga et al., 2024; Tong et al., 2025). Where applicable, core microbiome membership is often defined using quantitative prevalence and/or abundance thresholds (Ping et al., 2024; Gu et al., 2025). Commonly, taxa detected in ≥ 50–80% of samples within a given host, soil type, or cropping system are designated as core members, sometimes in combination with a minimum relative abundance cutoff (e.g., ≥ 0.1–1%). More stringent definitions restrict core taxa to those present across all sampled locations or time points, whereas relaxed thresholds capture conditionally persistent members (Amutuhaire et al., 2025). However, thresholds remain study-specific and are influenced by sequencing depth, taxonomic resolution, and sampling design. Accordingly, transparent reporting and context-dependent justification of core criteria are essential for cross-study comparison and downstream applications such as synthetic community design (Alzate Zuluaga et al., 2024). The concept of a “core microbiome” has been widely applied to identify microbial taxa consistently associated with host plants or soils; however, accumulating evidence indicates that core membership is highly contingent on soil type, climate, and cropping system. Edaphic factors such as pH, texture, organic matter, and nutrient availability strongly shape fungal community assembly, while climatic variables including temperature and precipitation impose additional selective pressures (Tong et al., 2025). Consequently, taxa considered core in one agroecosystem may be absent or functionally redundant in another. This variability challenges the transferability of microbiome-based interventions and highlights the need to contextualize core mycobiomes across environmental gradients. Systematic comparison of core fungal taxa across soil types, climates, and major crop systems is therefore essential to inform the rational design of synthetic communities with ecological relevance and functional stability (Wu D. et al., 2024).

**TABLE 1:** Variability in reported core fungal taxa across soil types, climates, and crop systems.

System/ context	Soil type/climate	Crop system	Reported core fungal taxa (examples)	Dominant functional roles	Key reference(s)
Temperate agroecosystems	Loamy, neutral pH; temperate climate	Wheat, barley	*Fusarium*, *Mortierella*, *Cladosporium*	Saprotrophy, nutrient cycling	(Pradhan et al., 2025)
Tropical systems	Acidic, highly weathered soils; humid tropics	Rice	*Glomeromycota* spp., *Trichoderma*	Mycorrhizal symbiosis, biocontrol	(Chen Q. et al., 2024)
Arid/semi-arid systems	Sandy soils; low rainfall	Maize, sorghum	*Aspergillus*, *Penicillium*	Stress tolerance, phosphate solubilization	(Gu et al., 2025)
High-input conventional farming	Fertilized, disturbed soils	Oilseed rape	*Alternaria*, *Epicoccum*	Opportunistic colonization	(Ping et al., 2024)
Low-input/organic systems	High organic matter soils	Vegetables	*Mortierella*, *Chaetomium*	Decomposition, soil health	(Amutuhaire et al., 2025)

### 2.2 Plant–microbe signaling networks (root exudates, quorum sensing, phytohormones)

Plant–microbe signaling in the rhizosphere forms a dense, dynamic communication network in which root exudates, microbial quorum-sensing molecules and phytohormones collectively coordinate microbial assembly, activity and plant physiological responses, a multilayered language that determines whether the rhizosphere becomes supportive or antagonistic under environmental stress (Qiao Y. et al., 2024). Root exudates (sugars, amino acids, organic acids, secondary metabolites, and specialized signals such as strigolactones) serve as nutritional cues as well as selective signals to recruit, stimulate, or repel particular bacterial, fungal, and protistan taxa, actively regulating community composition and functional potential (Wu D. et al., 2024; Yang et al., 2025). Microbes in response use quorum sensing (AHLs, oligopeptides, and analogous signals) in coordinating biofilm formation, secondary-metabolite production, and colonization behaviors that adjust plant access to nutrients and defense outcomes; and plants can perceive, mimic, or disrupt these signals (quorum-quenching) to bias microbiome activity (Majdura et al., 2023; Zheng et al., 2025). Phytohormonal interactions with auxin, ethylene, jasmonic acid, salicylic acid, and microbially produced hormones and hormone-modifying enzymes (e.g., ACC deaminase) form bidirectional signaling networks: microbes modify root structure and stress response through hormone availability or regulation, and plant hormonal status responds through altered exudation patterns and microbial recruitment (Orozco-Mosqueda et al., 2023; Schmidt et al., 2024). Recent metabolomics and multi-omics analyses have found that these signals work across both the kingdoms (bacteria, fungi, archaea, and protists) and spatial scales, yielding context-dependent emergent properties, such as pathogen attack or drought, which can rapidly reprogram exudation to recruit protective taxa, and microbial VOCs and QS-regulated metabolites can systemically prime plant immunity (Tong et al., 2025; Uribe-Acosta et al., 2025). Synthetic microbiomes and bioengineered rhizospheres require signal specificity to convert synthetic microbiomes and bioengineered rhizospheres. Signal specificity (what metabolites elicit what responses), time dynamics (when signals are produced), and cross-kingdom signal integration will be required: engineering Effective SynComs will involve linking keystone signal producers with host genotypes and soil environments to form robust signal networks that sustain nutrient provisioning and stress resilience during field conditions (Wu D. et al., 2024; Pradhan et al., 2025). The functional requirements imposed on microbiomes vary widely depending on the ecological and agronomic context (Zheng et al., 2025). In some cases, a single dominant function such as pathogen suppression or nutrient mobilization may be sufficient to confer measurable benefits to the host. In contrast, many agricultural systems require the coordinated delivery of multiple functions, including nutrient acquisition, abiotic stress tolerance, growth promotion, and disease resistance (Schmidt et al., 2024). These multifunctional demands often exceed the capacity of individual microbial taxa, underscoring the need for multi-member synthetic communities that integrate complementary functional traits. The degree to which core microbiomes have been defined varies substantially among crop species, largely reflecting differences in research intensity, economic importance, and experimental tractability. Major staple crops such as wheat, rice, and maize possess relatively well-characterized core microbiomes, with multiple studies consistently reporting dominant bacterial and fungal taxa across diverse soils and geographic regions (Orozco-Mosqueda et al., 2023). In contrast, the core microbiomes of many horticultural crops (e.g., leafy greens), legumes beyond model systems, and perennial or orphan crops remain poorly defined, with limited cross-site validation and inconsistent taxonomic resolution. An additional consideration in defining core microbiomes is the temporal stability of constituent taxa. While some core fungal members persist across plant developmental stages, growing seasons, and years, others exhibit transient or context-dependent associations driven by phenology, climate variability, and management practices. As a result, taxa identified as “core” based on single time-point sampling may not represent stable or functionally consistent community members (Orozco-Mosqueda et al., 2023; Yang L. et al., 2024; Uribe-Acosta et al., 2025). Incorporating temporal replication is therefore critical for distinguishing persistent core taxa from opportunistic or seasonally enriched fungi. Microbial signaling pathways, including quorum sensing, secondary metabolite production, and cross-kingdom communication, are highly sensitive to environmental conditions. Soil physicochemical properties such as pH, moisture, temperature, and nutrient availability can enhance or suppress signal molecule production, alter diffusion rates, and affect receptor sensitivity (Majdura et al., 2023; Yang et al., 2025; Zheng et al., 2025). Climatic variables including seasonal temperature fluctuations, precipitation patterns, and light exposure further modulate microbial activity and signaling dynamics. Additionally, management practices such as fertilization, irrigation, and crop rotation can reshape the soil microenvironment, indirectly influencing microbial communication and the stability of functional interactions. These context-dependent effects underscore the importance of considering environmental modulation when designing synthetic communities for reproducible and robust functional outcomes (Orozco-Mosqueda et al., 2023).

### 2.3 Ecological drivers shaping rhizosphere communities

**FIGURE 1:**
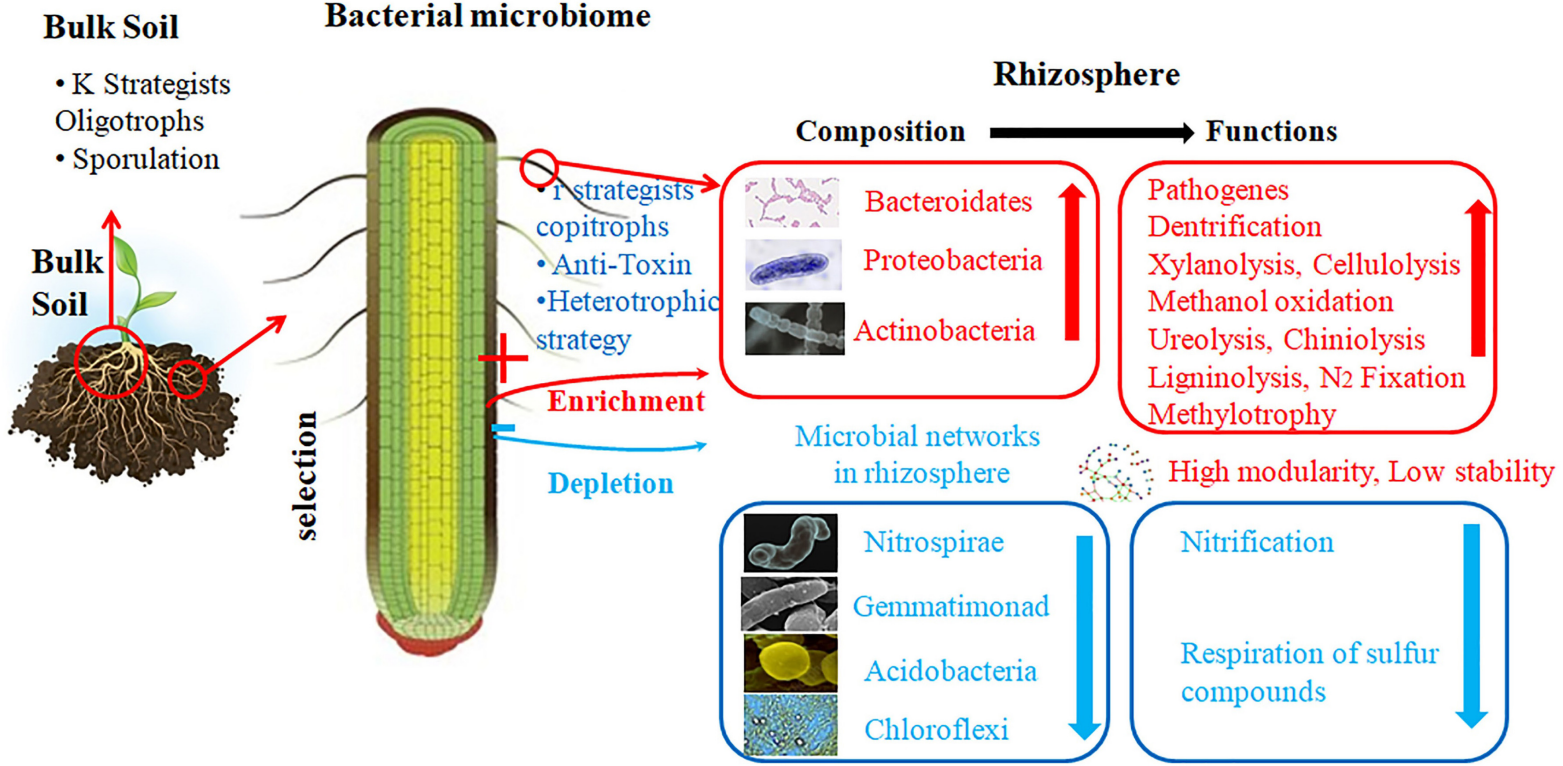
Schematic of rhizosphere-mediated selection of soil bacterial communities. Root exudates enrich copiotrophic and heterotrophic taxa (Bacteroidetes, Proteobacteria, Actinobacteria) and associated functions including decomposition of carbon, denitrification, N2 fixation, and methylotrophy while depleting oligotrophic groups (Nitrospirae, Acidobacteria, Chloroflexi) linked to nitrification and sulfur respiration. These changes yield highly modular but less stable microbial networks in the rhizosphere compared with bulk soil.

The hierarchy of ecological drivers influences the composition of rhizosphere communities and operates on the spatial and temporal levels to produce predictable filters and situation-specific outcomes, in contrast to the simple deterministic assemblages (Figure 1 and Table 2). Microsite-scale abiotic filters (physicochemical properties of soil, pH, texture, and redox status), moisture and temperature conditions, and nutrient supply create the template of the baseline habitat that constrains the kind of taxa and functional features that can be supported and metabolized within the root zone (Yang et al., 2025). Beyond these abiotic thresholds are biotic filters, which are driven by biological aspects of the plant– root structure, exudation chemistry, and phenology impose resource landscapes and micro-niches that selectively enrich taxa of relative metabolic abilities or competitive strategies, and the developmental stage of the plant and genotype further modulates recruitment patterns (Majdura et al., 2023; Singh et al., 2025). Land-use and management history (tillage intensity, fertilization regime, crop rotation, cover crops, and organic amendments) have the ability to reorganize soil habitat and propagule pools to create alternative stabilizing states or directional changes in rhizosphere composition and functional potential, which can be resistant to change over a few seasonal cycles (Schmidt et al., 2024; Zheng et al., 2025). Periodic (temporal) dynamics, such as seasonality, interannual climate variability, and successional aging of the plant host, encourage recurrent turnover and pulses of activity that interact with dispersal and priority effects; early arriving microbes and transient blooms (e.g., after root flushes or rewetting of droughts) can have disproportionately long-term effects on community trajectory and ecosystem services (Orozco-Mosqueda et al., 2023). On a larger scale, the local landscape context and climate (precipitation patterns, extreme temperatures, altitude) define the pool of species available to the region and limit the variety of potential community compositions, so that the same host genotypes or SynCom introductions can often have divergent effects across sites (Yang et al., 2025). Notably, ecological interactions such as competition, facilitation, predation (protists, nematodes), and viral lysis mediate assembly to influence realized abundances and functional expression in a way beyond prediction by abiotic filtering. Trait-based and assembly-theory models (filter → interaction → drift) thus provide a more mechanistic foundation for predicting rhizosphere composition compared to taxon lists alone and identify key design levers to engineer rhizospheres: Adjust habitat filters (soil amendments, irrigation), adapt microbial functional traits to host exudate niches, and manage temporal windows (planting/cover crop timing) to take advantage of priority effects. Recognizing these multi-scale drivers, and their non-linear responses to extreme events such as drought or heat waves is essential for translating laboratory SynCom successes into durable, field-scale solutions for climate-resilient agriculture (Orozco-Mosqueda et al., 2023; Zheng et al., 2025).

**TABLE 2:** Key microbial signaling molecules and their functions.

Signal molecule/class	Microbial source	Primary function(s)	Environmental modulation	Representative references
Quorum-sensing molecules (e.g., AHLs, AI-2)	Bacteria	Regulate population density, biofilm formation, virulence factor expression	pH, temperature, nutrient levels, soil moisture	(Yang et al., 2025)
Volatile organic compounds (VOCs)	Bacteria and fungi	Inter-kingdom communication, plant growth promotion, pathogen suppression	Soil aeration, moisture, temperature	(Yang et al., 2025)
Auxins (IAA)	Bacteria and fungi	Modulate plant root architecture, promote growth	Nutrient availability, light, carbon source	(Majdura et al., 2023)
Siderophores	Bacteria and fungi	Iron acquisition, competition with pathogens	Iron availability, pH	(Majdura et al., 2023)
Fungal secondary metabolites (e.g., polyketides, terpenes)	Fungi	Antimicrobial activity, niche establishment, signaling	Temperature, nutrient levels, host presence	(Zheng et al., 2025)
Lipopeptides (e.g., surfactins, fengycins)	Bacteria	Biofilm formation, pathogen suppression	Moisture, nutrient levels	(Schmidt et al., 2024)
Mycorrhizal signaling molecules (e.g., strigolactones, Myc factors)	Plants and AMF	Symbiosis initiation, root colonization	Phosphate availability, host developmental stage	(Orozco-Mosqueda et al., 2023)

Among the diverse microbial signaling pathways, some are more tractable for engineering than others (Uribe-Acosta et al., 2025). Quorum-sensing molecules, for example, are relatively straightforward to manipulate through inoculation with signal-producing or signal-degrading strains, chemical analogs, or genetic modification of key synthases. Similarly, the production of auxins (IAA) or siderophores can be enhanced by selecting or engineering microbial taxa with high metabolite output, or by adjusting readily controllable environmental factors such as nutrient availability (Yang L. et al., 2024). Volatile organic compounds (VOCs) and fungal secondary metabolites offer functional versatility but are more sensitive to environmental variation, making their consistent deployment challenging in field conditions. Strigolactones and Myc factors, which mediate plant–fungal symbiosis, are partially host-driven and thus require integrated host–microbe engineering rather than microbial-only manipulation (Pang and Xu, 2024). Overall, signals with microbially autonomous production and limited environmental dependency are most amenable to targeted engineering in synthetic communities, while host- or environment-dependent signals demand more sophisticated or context-specific strategies.

## 3 Synthetic microbiomes in agriculture

A critical definition of synthetic microbiomes, also known as SynComs, is established for a multi-species membered microbe consortium to deliver greater functional stability and reproducibility than that of a single membered PGPR inoculum. The application of synthetic microbiomes includes a microbe consortium that is drought resilient and composed of Bacillus, Pseudomonas, Azospirillum, and mycorrhizal fungi that improve crop architecture, water efficiency, and stress response, alongside a microbe consortium that is capable of fixing free nitrogen and incorporates free living diazotrophs and nutrient solubilizing microbes to eliminate fertilizer requirements in non-legume crops (Bueno de Mesquita et al., 2024).

### 3.1 Approaches to microbiome assembly

#### 3.1.1 Top-down (selective enrichment)

Top-down (selective enrichment) strategies steer native rhizosphere communities toward desirable functional states by manipulating environments, hosts or resource landscapes to favor pre-existing beneficial assemblages rather than assembling microbes *de novo*. Practically, this includes field-scale interventions (crop rotation, cover crops, organic amendments, reduced tillage) that condition soil and root exudation profiles to enrich disease-suppressive or nutrient-mobilizing consortia, as well as laboratory and greenhouse serial-passage or substrate-selection experiments that compress community complexity while preserving key interactions (Yang L. et al., 2024; Uribe-Acosta et al., 2025). Selective enrichment can be implemented *in situ* (management-driven conditioning) or *ex situ* (microcosm passaging and in-planta enrichment) to isolate stable, high-performing consortia, recent in-planta enrichment pipelines have successfully recovered “super” endophytes and simplified consortia that retain multifunctionality when reintroduced to hosts (Bueno de Mesquita et al., 2024; Pang and Xu, 2024). The major strengths of the top-down approach are ecological realism (maintenance of emergent interspecies interactions and trophic links), scalability for field application, and the ability to exploit local adaptation and resident propagule pools; these qualities make it especially attractive for engineering rhizospheres to withstand abiotic extremes such as drought or nutrient stress (Jing et al., 2023; Seitz et al., 2024). Yet top-down enrichment faces important constraints: outcomes are highly context-dependent (soil legacy, climate, host genotype), enriched functions may be transient without continued selective pressure, and mechanistic attribution of effects is difficult without coupling to multi-omics and reductionist follow-ups (Jing et al., 2023; Yang L. et al., 2024). Current best practice therefore integrates top-down conditioning with targeted bottom-up reconstruction and omics-guided monitoring, using enrichment to reveal robust, locally adapted guilds and then rationalizing those guilds into tractable SynComs or management prescriptions that aim for durable, climate-resilient agronomic benefits (Jing et al., 2023; Seitz et al., 2024).

#### 3.1.2 Bottom-up (rational design with defined consortia)

**FIGURE 2:**
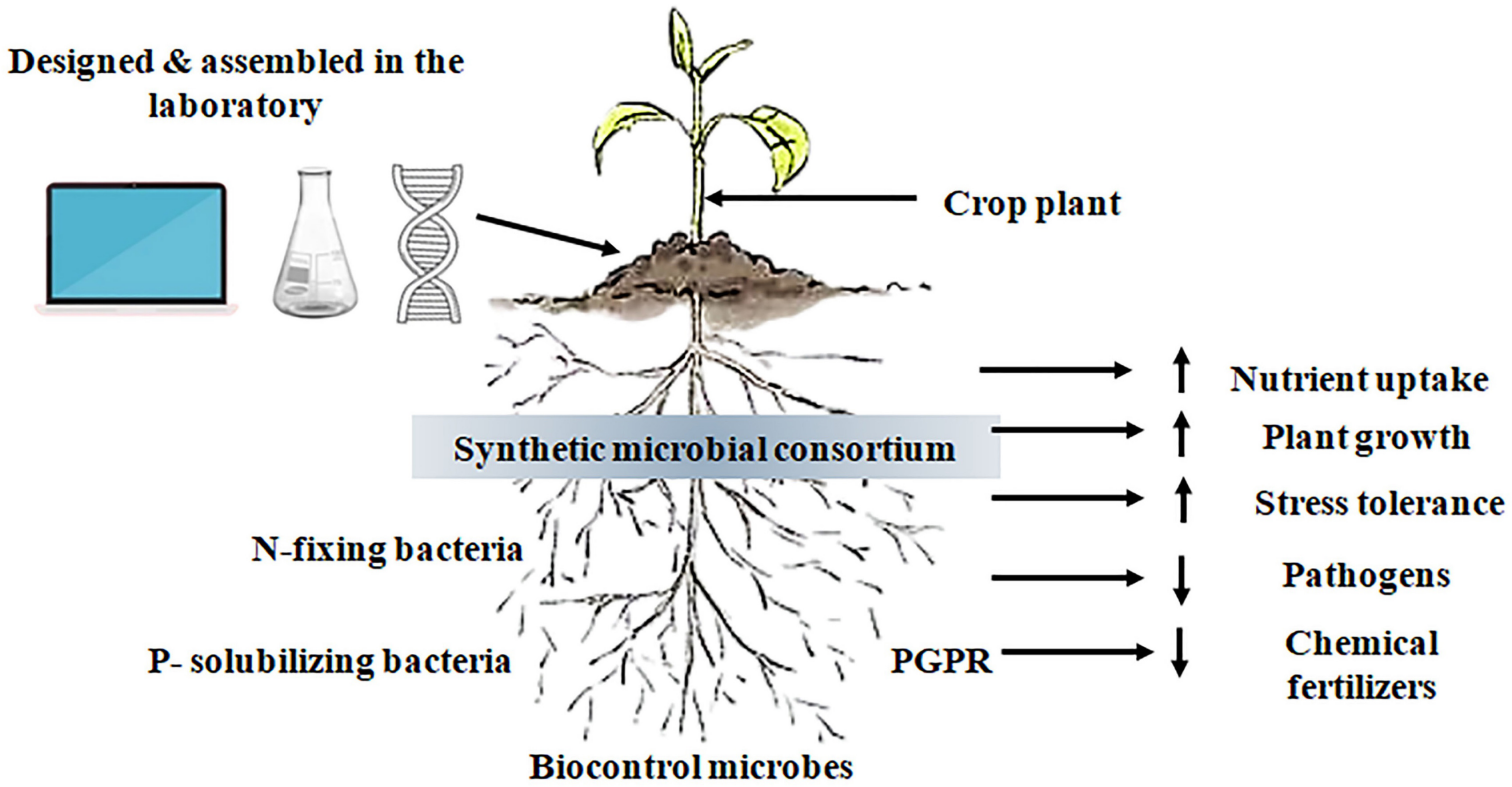
Conceptual framework of a synthetic microbial consortium for plant-soil interaction studies. The figure shows how computational design, laboratory assembly, and genetic insight together create a synthetic microbial consortium to be targeted to the plant rhizosphere. *In silico* tools and data-driven approaches enable the selection of microbial members, which are then cultured and assembled under controlled conditions in the laboratory.

Bottom-up, rational design of defined consortia constructs plant-associated microbiomes from the ground up by selecting and combining strains with complementary metabolic capabilities, interaction phenotypes and ecological roles to produce predictable functions and stability in the rhizosphere (Figure 2 and Table 3). Recent frameworks emphasize modularity, building networks of functionally coherent “modules” (e.g., nutrient-mobilizers, hormone producers, antagonists, and biofilm formers) that can be mixed and matched to match host genotype and soil context, rather than assembling large, *ad hoc* mixtures (Schmidt et al., 2019). Predictability is strengthened by coupling high-throughput pairwise interaction assays and microcosm co-cultures with genome-scale metabolic models (GEMs) and constraint-based community simulations that identify cross-feeding links, metabolic auxotrophies and potential competitive bottlenecks prior to in-planta testing (Zhou et al., 2023; Argiroff et al., 2024). Engraftment is a fundamental practical goal: designed consortia are selected on the basis of colonization properties, stability in the face of resident microbiota, and the capacity to recruit or rewire host reactions, which are increasingly being evaluated in greenhouse and field contexts where small, well-characterized strains outperform larger, less characterized mixtures (Lyu et al., 2024; Khouri Chalouhi et al., 2025). Ecological design principles (keystone strain positioning, redundancy of essential functions, and the existence of helper taxa that stabilize interactions) and the design-build-test process enable the performance and persistence to be fined and discover failure modes, including invasion resistance or context-dependent loss of function (Ruan et al., 2024; Jourdain and Gu, 2025). Finally, to achieve such translational readiness, standardized pipelines to phenotyping, regulatory foresight, and approaches to scale production and formulation without degrading functionality are needed, which are being addressed in the current literature through modular, model-guided solutions and the focus on minimal, well-characterized consortia that are both controllable and ecologically realistic (Granato et al., 2019; Schmidt et al., 2019).

**TABLE 3:** Comparing bottom-up and top-down methods for the assembly of synthetic microbiomes.

Parameter	Top-down (selective enrichment)	Bottom-up (rational design with defined consortia)	Advantages	References
Microbes source	Begins with a natural complex microbial community (e.g., soil, rhizosphere, gut) and enriches beneficial members	Uses individually isolated or engineered microbial strains selected based on known traits/functions	Top-down: retains natural diversity; bottom-up: high precision and control	(Yang L. et al., 2024)
Environmental stability	Generally high due to preserved native interactions within the community	May be lower initially as designed consortia need adaptation to natural environments	Top-down systems show better resilience and robustness in real environments	(Pang and Xu, 2024)
Design flexibility	Limited—community composition shaped by environmental selection and enrichment steps	High level—microbes are intentionally selected, combined, or engineered for specific functions	Bottom-up allows targeted functional design	(Bueno de Mesquita et al., 2024)
Required time and effort	Faster initial establishment since nature performs selection	More time-consuming due to isolation, characterization, and compatibility testing	Top-down is time-efficient; bottom-up yields more customized solutions	(Pang and Xu, 2024)
Mechanistic understanding	Harder to dissect due to unknown species and interactions	Easier to study due to known and characterized members	Bottom-up supports hypothesis-driven research	(Yang L. et al., 2024)
Application suitability	Best for ecological restoration, soil/rhizosphere improvement, and field-ready microbiomes	Best for targeted applications like nitrogen fixation, stress-tolerance, disease suppression, or metabolite production	Both approaches complement each other depending on task	(Bueno de Mesquita et al., 2024)

### 3.2 Criteria for microbial selection (compatibility, functionality, stability)

Critical microbial selection necessary to produce a successful SynCom must be an integrative, evidence-based rubric of three interdependent criteria, including compatibility, functionality, and stability with the ecological realities of the target field environment. The notions of compatibility will be assessed outside the context of non-antagonism: strains will be compatible through pairwise and small community experiments, not form antagonistic toxins to the members of the consortium, and its niche preference will be complementary (colonization site, oxygen tolerance, substrate usage) to minimize competitive exclusion; high-throughput interaction screens and coexistence modeling are operable methods to quantify such attributes (Chen X. et al., 2024; Spooren et al., 2024). Functionality requires clear, measurable mechanisms tied to the desired agronomic outcome (e.g., N-fixation rates, phosphate-solubilization capacity, IAA or ACC-deaminase production, siderophore-mediated micronutrient mobilization, or pathogen antagonism); pairing phenotypic assays with genomic markers and genome-scale metabolic models permits both confirmation of activity and prediction of cross-feeding or metabolic redundancy across strains (Giordano et al., 2024; Hsieh et al., 2024). Stability, the capacity to engraft, persist and perform under fluctuating field conditions, is a function of ecological fit (matching to the resident soil community and host genotype), life-history traits (spore formation, biofilm/adhesion factors), and network position (keystone or helper roles that buffer against invasion or loss); stability should therefore be quantified with time-series engraftment experiments, stress-challenge assays (drought, heat, nutrient shocks) and network-level analyses that identify resilience and critical taxa (Jing et al., 2023; Zhou et al., 2024). Practical selection pipelines integrate these criteria iteratively: (i) screen isolates for target traits and colonization phenotypes, (ii) map pairwise and higher-order interactions to filter incompatible combinations, (iii) use metabolic and ecological models to predict emergent behaviors, and (iv) validate engraftment and functionality in progressively realistic hosts/soils with multi-omics readouts to capture active expression rather than mere presence (Sun et al., 2024; Zhou et al., 2024). Finally, translational success depends on representation of functional redundancy for key services, inclusion of “helper” taxa that facilitate engraftment, and attention to formulation and delivery (carriers, protectants, timing) that preserve viability and ecological effectiveness, criteria that together move SynCom design from promising proof-of-concepts toward durable, climate-resilient agricultural applications (Granato et al., 2019; Durán et al., 2025).

The success of rhizosphere bioengineering depends not only on selecting beneficial microbial strains but also on ensuring their long-term viability, stability, and functional consistency. Effective strain preservation strategies are essential to maintain inoculum integrity during storage, transport, and large-scale deployment. Common preservation methods include cryopreservation at -80°C, lyophilization (freeze-drying), encapsulation in protective carriers, and storage in glycerol or other cryoprotectants. Each method has trade-offs in terms of cost, viability retention, and ease of field application. Equally important is the implementation of rigorous quality control measures. These include regular assessment of microbial identity using molecular markers (e.g., sequencing or qPCR), monitoring functional traits (e.g., phosphate solubilization, antifungal activity), and verifying absence of contaminants. Quality control ensures that engineered or synthetic consortia maintain their designed functionality across batches and over time, a critical requirement for reproducibility and regulatory compliance. For multi-strain consortia, compatibility and interaction stability should be monitored during storage and prior to field application. Overall, integrating robust preservation protocols with systematic quality control not only enhances the reliability and scalability of rhizosphere bioengineering but also builds confidence for adoption in agriculture under variable environmental and regulatory conditions.

The use of genetically modified microorganisms (GMMs) in the rhizosphere offers promising opportunities for precision microbiome engineering, but it also carries potential ecological risks. One key concern is horizontal gene transfer, where introduced genetic elements may spread to native soil microbes, potentially altering microbial community dynamics or generating unintended metabolic capabilities. Another risk is ecological disruption; engineered strains may outcompete native taxa, reduce biodiversity, or interfere with existing microbial networks, with consequences for nutrient cycling and soil health. Environmental persistence and proliferation beyond target sites also pose a concern, particularly under variable field conditions such as fluctuating temperature, moisture, or nutrient availability.

Risk assessment must therefore consider both the traits of the engineered strain and the characteristics of the receiving environment. Strategies to mitigate risks include the use of biological containment systems (e.g., auxotrophy, kill switches), thorough ecological fitness testing, and controlled field trials with ongoing monitoring. Regulatory frameworks in many countries require detailed documentation of potential environmental impacts before GMM release, emphasizing the need for careful stewardship and robust ecological evaluation.

### 3.3 Case studies of synthetic consortia improving drought, salinity, and pathogen resistance

Synthetic consortia assembled from well-characterized strains are increasingly shown to confer meaningful tolerance to drought, salinity and pathogens by combining complementary mechanisms (osmoprotection, root architecture modulation, ion homeostasis, antibiosis and induced systemic resistance) into single, deployable units, an approach now supported by both targeted experiments and synthesis reviews (Jing et al., 2024). Drought-focused case studies illustrate this potential: for example, paired inoculation of Azotobacter chroococcum with Trichoderma afroharzianum improved tomato productivity under a 30% water reduction and increased yield and resilience metrics in controlled trials, while trait-informed SynComs in recent reports have been shown to enhance drought survival and water-use efficiency across cereals and tree seedlings (Zhu et al., 2024; Pineda-Pineda et al., 2025). Salinity resilience has been achieved by mining salt-adapted microbiomes and translating those taxa into consortia—studies of salt-tolerant, mangrove-derived communities and engineered Bacillus consortia demonstrate improved seedling growth and ionic balance in saline soils, often through combined mechanisms of osmolyte production, exopolysaccharide-mediated rhizosphere buffering and siderophore-driven micronutrient acquisition (Velte et al., 2025; Wang et al., 2025). In disease control, multi-strain mixes have been shown to be superior to single strains in suppressing greenhouse pathogens: synthetic bacteria and mixed bacterial-fungal communities elicit less severe symptoms and altered resident microbiomes toward suppressive phenotypes in crops like apple and other horticultural hosts, which demonstrates how complementary antagonists and recruitment assists can create long-term biocontrol effects (Jing et al., 2024). Notably, translational case studies that compare winter-crop field trials and multi-season microcosm enrichment report both successes and constraints: consortia can establish and deliver services in context, yet performance is highly place- and heritage-dependent, requiring screening of pairwise interaction, redundancy of essential services, and adaptive delivery/formulation to achieve engraftment under field variation (Jing et al., 2024; Durán et al., 2025). All of these case studies collectively indicate a scalable, trait-guided SynCom design that pairs stress-adaptive strains (through extremophile or locally adapted pools) with helper taxa that improve colonization and network stability, an architecture that implies a plausible practical path to a scalable, climate-resilient microbiome intervention and also indicates the value of rigorous field validation and mechanistic multi-omics to associate taxa to function (Jing et al., 2024; Durán et al., 2025).

While genetically modified microorganisms (GMMs) hold promise for precise manipulation of rhizosphere functions, their application faces significant ecological and practical challenges. Beyond the potential benefits, GMMs can disrupt native microbial communities through competition or unintended metabolic interactions, possibly altering nutrient cycling, pathogen suppression, or soil health (Sun et al., 2024). Horizontal gene transfer is a major concern, as engineered traits may spread to non-target soil microbes, with unknown ecological consequences. Environmental variability including temperature fluctuations, moisture stress, and nutrient limitations can reduce the survival and functional reliability of engineered strains, limiting their consistency across field conditions. Furthermore, establishing stable colonization and interaction with plants or soil matrices remains technically challenging, especially for multi-strain consortia (Durán et al., 2025).

Regulatory and public acceptance hurdles add additional layers of complexity. Many countries impose stringent requirements for field release of GMMs, including detailed environmental risk assessments, containment strategies, and long-term monitoring. Combined with high costs of strain development, scale-up, and quality control, these challenges mean that translating engineered rhizosphere microbes from the lab to the field is far from straightforward. Recognizing these limitations is crucial to set realistic expectations and guide the development of safe, effective, and context-specific microbiome interventions (Jing et al., 2024). For contexts where genetically modified microorganisms (GMMs) face regulatory, ecological, or public acceptance barriers, non-GMO approaches such as adaptive evolution and selective enrichment offer viable alternatives (Carrión et al., 2019). Adaptive evolution involves exposing microbial strains or communities to defined selective pressures (e.g., drought, salinity, or nutrient limitation) over multiple generations to enhance desired traits, such as stress tolerance, nutrient acquisition, or metabolite production (Zhu et al., 2024; Pineda-Pineda et al., 2025; Velte et al., 2025). Similarly, selective enrichment of naturally occurring beneficial microbes from soil or rhizosphere samples can increase the abundance of functionally relevant taxa without genetic modification. These approaches retain naturally evolved regulatory networks, maintain ecological compatibility with native microbial communities, and generally face fewer regulatory constraints (Jing et al., 2024; Wang et al., 2025). While adaptive evolution may require extended cultivation periods and careful monitoring to prevent unintended trade-offs, it represents a pragmatic strategy for improving microbial performance *in situ*, especially in agriculture and environmental applications where GMO deployment is limited (Jing et al., 2024).

## 4 Bioengineering the rhizosphere

Rhizosphere bioengineering-meaning strategic manipulation of beneficial microbes through advanced genetic technologies-is one of the strong frontiers in modern agriculture and environmental management and brings together synthetic biology with targeted ecosystem enhancement. Basic to the genetic engineering of rhizosphere microorganisms is the intent to improve the interaction between plants and soil by giving the microorganisms improved or completely new traits, thus enhancing functionality in plant growth, nutrient cycling, and stress tolerance (Tariq et al., 2025). Nitrogen fixation, or the process whereby atmospheric nitrogen is converted into ammonium, is another major focus; through transferring nitrogenase enzyme gene clusters or optimizing regulatory elements using CRISPR-based genome editing, several bacterial strains have been generated displaying increased nitrogenase activities, hence lowering crop reliance on synthetic fertilizers and therefore improving sustainability (Shayanthan et al., 2022). Such improved microbial nitrogen fixers have now shown measurable increases in plant productivity under field conditions, representing translation from laboratory success to agricultural benefit. Phosphate solubilization is another target for rhizosphere bioengineering because of its immobility and poor bioavailability in soil (Cirillo et al., 2023). It is possible, with both the conventional transformation and CRISPR-Cas systems, to increase the expression of genes encoding organic acid production, phytase, and other enzymes responsible for releasing phosphate ions from insoluble complexes. Such microbes not only enhance nutrient use efficiency in crops but also contribute toward soil remediation affected by excessive fertilizer use. Beyond macronutrients, engineering stress-responsive metabolites in rhizosphere microbes is considered a newer frontier for facilitating the adaptation of plants to hostile environments that include salinity, drought, and pathogen pressure. For instance, researchers have endowed bacteria with the machinery to protect host plants directly through biochemical support or indirectly by modulating defense signaling networks by enhancing the synthesis pathways toward osmoprotectants, antioxidants, or antifungal compounds. Genetic changes are made with precision, often using multiplexed CRISPR technology, which enables the simultaneous fine-tuning of several metabolic genes involved in optimizing the microbial response to real-world variable soil stress factors (Mikiciuk et al., 2024). The manipulation of manufactured root exudates to shape and regulate the rhizosphere microbiome’s composition is equally essential to the bioengineering vision. Numerous organic compounds that serve as both complex nourishment and messenger molecules for communities of bacteria are naturally released into the soil by plants (Yang X. et al., 2024). By creating and expressing new exudate characteristics in bacteria connected to roots, synthetic biology can improve the microbiome’s structure or function. In addition to increasing the resilience of microbial communities and suppressing pathogenic species, this promotes synergistic effects that lead to strong multi-trait microbial communities for specific soil types and crops. In this sense, CRISPR-based techniques have revolutionized microbial genome engineering by offering multiplexed, efficient, and targeted gene modification in both experimental and non-model organisms. CRISPRi and base-editing systems have been combined with deactivating proteins known as Cas or high-fidelity nucleases to enable precise base changes and reversible gene activation or silencing without causing broken double-strands or requiring biological templates (Zhang et al., 2024). The capacity for growth of rhizosphere engineering endeavors has improved thanks to complementary approaches that have improved current delivery systems, created synthetic directional RNA, and created algorithms that predict for gene editing (Qiao R. et al., 2024). As a result, this makes it possible to optimize metabolic pathways and create microbial libraries with a variety of traits through multiplex CRISPR applications, which enable quick screening for high-performance strains in a range of soil conditions. The genetic manipulation of *Pseudomonas* species and Corynebacterium strains for improved phosphate dissolution as well as the manufacturing of vitamin pantothenic acid using CRISPR-Cpf1 and RecET-mediated editing techniques are examples of effective bioengineered rhizosphere interventions. These enhanced characteristics supported their potential as efficient biofertilizers and biostimulants by having a positive effect on yield, nutrient soil accessibility, and genetic manipulation (Behr et al., 2023). Similarly, CRISPR has made it possible to unlock previously dormant biosynthetic gene clusters in the Streptomyces genome for the discovery of natural products, thereby increasing the toolkit against crop-related insects, diseases, and abiotic stressors. Due to improved delivery techniques, stable editing complex expression, and reduced off-target effects, type I and second-generation CRISPR systems further expand the reach and enable gene editing in previously resistant microbial strains. Fine-tuning microbial metabolism in conjunction with base editor and CRISPR transcription platforms enables the development of “creative” probiotics and consortia that are especially tailored for rhizosphere applications (Dwivedi et al., 2025). The possibility of completely programmable roots microbiomes is very close thanks to the expanding collection of engineered regulatory networks, genome-scale biological models, and artificial intelligence-driven design guidelines made possible by CRISPR and related technologies (Ke et al., 2021). However, the application of artificially produced organisms in the rhizosphere will need to strike a balance between innovation, safety issues, concerns about the environment, and regulatory frameworks given these encouraging prospects (Enespa and Chandra, 2022). In light of the possible dangers of gene transfer into organisms that are not targets or unintentional disruptions to ecosystems, the moral framework is still being developed, and prevention, surveillance, and risk assessment procedures are still being refined. In conclusion, an entirely novel form of smart agriculture based on effective nutrient cycling, improved stress tolerance, and precise microbiome management is being produced by bioengineering the root system through modification of genes and CRISPR-driven microbial manipulation (Yang and Wang, 2025). Hence, these novel measures, already being scrutinized through laboratory and pilot plant experiments, are viewed as a bet on future management of the ecosystem, food supply, and technology with trust in the latter (Yang and Wang, 2025).

## 5 Plant–microbe interactions under climate stress

### 5.1 Effects of heat, flooding, salinity, and drought on rhizosphere microbiota

Rhizosphere, the very thin layer of organic matter that is impacted by plant roots, is the place where complex interactions between microorganisms and plants occur (Lu et al., 2025). Environmental factors such as moisture, extreme heat, and flooding very much influence the diversity of microbes in this region and their functions since it is a very sensitive area to environmental changes. The main problem in agriculture is drought stress. It reduces soil moisture and nutrient availability, which in turn results in modification in the composition and activity of microbial communities (Lu et al., 2025). During dry seasons, spore-producing and stress-resistant organisms might outnumber, while beneficial organisms like nitrogen-fixing and phosphate-dissolving ones are often reduced in their population. Changes of this nature negatively affect plant growth because the bacterial support for hormone production and nutrient uptake also goes down (Tamrakar et al., 2025).

**FIGURE 3:**
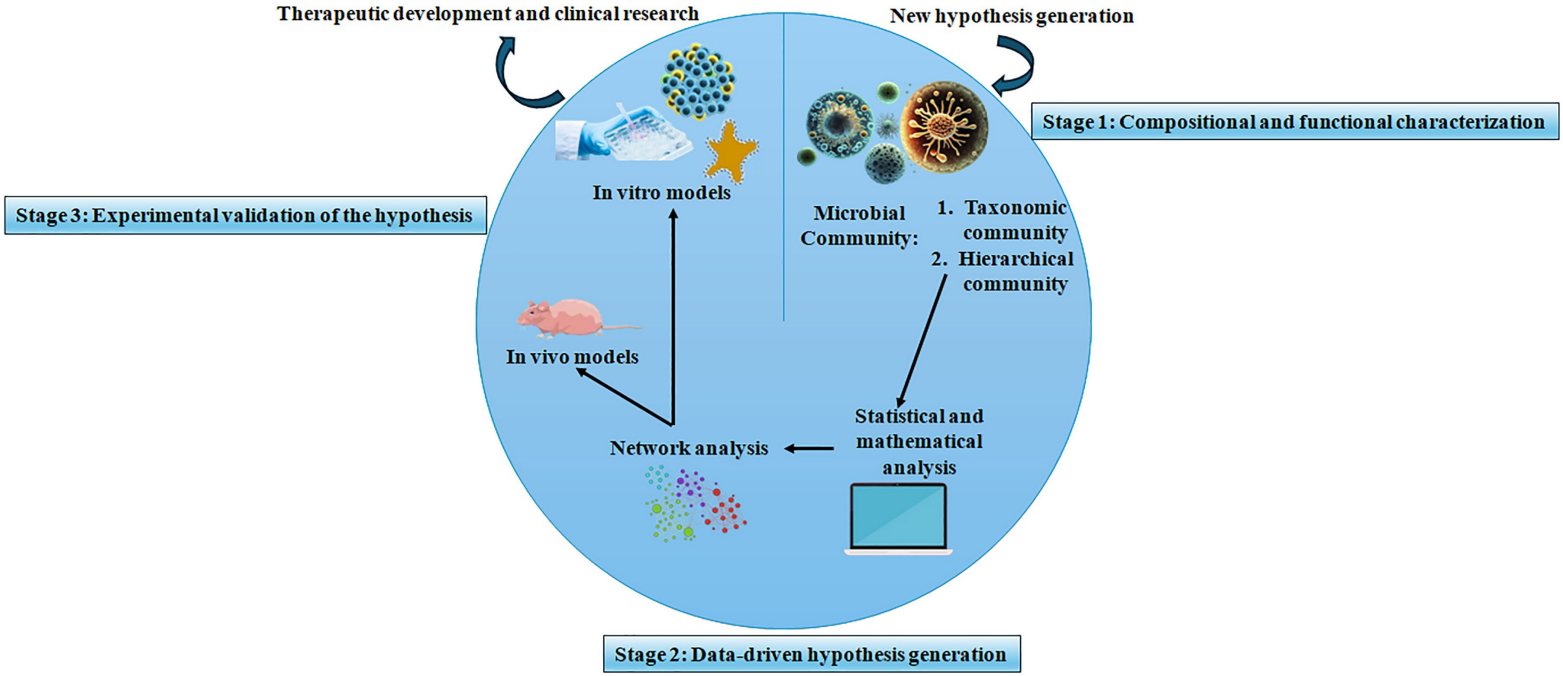
Role of beneficial plant-associated microbiomes in abiotic and biotic stress mitigation and growth promotion. The scheme compares the absence and presence of PGPMs, such as phyllosphere and rhizosphere-colonizing PGPR and AMF, which enhance plant tolerance to abiotic and biotic stresses, improve nutrient use efficiency and root health, and trigger induced systemic resistance, leading to enhanced growth and resilience under adverse environmental conditions.

Salinity stress leads to osmotic disturbances and ion toxicity which are detrimental to the rhizosphere microbiome (Kaiser et al., 2023). The rise in the amounts of sodium and chloride ions results in less microbial variety and also slows down the growth of bacteria and fungi that are susceptible to the conditions. Consequently, activities of microbes such as nitrogen fixation, fertilization, and organic matter decomposition are hindered (Roghair Stroud et al., 2024). However, some bacteria that are tolerant to the high levels of salt or even resistant to it, and also the plants that grow in such conditions, can produce sulfur-based compounds or exopolysaccharides that help improve the soil structure and reduce the salt stress on the roots of the plants (Figure 3).

Microbial interventions often improve plant and soil stress responses up to moderate levels of abiotic stress, but there appear to be severity thresholds beyond which these benefits are greatly diminished or lost (Yang and Wang, 2025). For example, soil microbial activity such as hydrogen uptake by key bacterial guilds drops sharply below water potentials of approximately -hydto -100 MPa, indicating a critical moisture threshold for basic microbial metabolism under drought conditions; below this range microbial activity may be effectively stalled, limiting rhizosphere functions regardless of inoculation efforts (Tamrakar et al., 2025). Similarly, exposure to high-intensity drought can trigger abrupt and persistent shifts in community structure and function reducing diversity and impairing soil microbial functioning even after moisture is restored, suggesting a threshold beyond which community resilience and recovery are compromised. Although microbial inoculants can mitigate stress impacts (e.g., enhancing antioxidant systems or osmolyte accumulation under drought or salinity), their capacity to compensate diminishes as stress severity increases past species-specific tolerance limits, such as very high salinity or extreme desiccation. Therefore, identifying and quantifying context-specific stress thresholds remains critical for realistic expectations of microbial engineering, highlighting that interventions are most effective within a moderate stress window beyond which engineered or selected microbes may be overwhelmed by environmental constraints (Kaiser et al., 2023). Heat stress is one more climate change effect that has a momentous impact on the microbes and plant elements of the rhizosphere. Increased temperatures stimulate microbial metabolism, which may ultimately lead to a reduction in bacterial biomass and a rapid carbon loss from organic matter (Roghair Stroud et al., 2024; Ma J. et al., 2025). Heat-tolerating microbes may die out, thus reducing overall diversity and disturbing the benefit of plant-microbe interaction by exposing plant roots to the harsh environment. Just like heavy rain alters the soil’s oxygen balance, creating conditions that favor anaerobic bacteria, the death of aerobic microorganisms results in earthy odors due to sulfur compounds (El-Saadony et al., 2024; Ma J. et al., 2025). Besides, the soil’s oxygen balance will be tipped in favor of anaerobic microorganisms which will eventually lead to methane production and denitrification. Soil microorganisms’ active metabolism will also be the case in such high temperature conditions where carbon dioxide is given off as a by-product during the process of decomposing organic matter. The combination of these different climate stresses creates a situation where the conditions for the natural balance of soil fertility and plant health are harder to maintain (Basu et al., 2021; El-Saadony et al., 2024; Ma J. et al., 2025).

### 5.2 Role of synthetic microbiomes in enhancing plant resilience

A practical biotechnological strategy to lessen the detrimental effects of climate stress on plant-microbe interactions is the development of synthetic microbial communities (Ramasamy and Mahawar, 2023). A synthetic microbiome is an artificially produced collection of beneficial microorganisms that collectively improve the development of plants, absorption of nutrients, and stress tolerance (Ramasamy and Mahawar, 2023; Ajuna et al., 2024). Unlike natural microbial communities, which develop naturally, synthetic microbiomes are designed with a specific advantageous goal in mind, such as increasing drought resilience or increasing the absorption for nutrients under salinity stress. Bacteria that are capable of produce exopolysaccharides, also called osmoprotectants, as well as abscisic acid (ABA), which aid vegetation in retaining water and maintaining turgor in drought-prone environments, can be found in synthetic microbiomes (El-Saadony et al., 2024; Roghair Stroud et al., 2024; Ma J. et al., 2025). To the extent that they might promote root lengthening and increase the efficiency of water usage, yeast Pseudomonas fluorescence, and the subtilis Bacillus have been added to produced microbiota. Microscopic-biomes of salt tolerating organisms like *Azospirillum* species or Halomonas strains can overshadow salt toxicity in salinity stress condition by controlling ion balance and producing IAA, which leads to better nutrient absorption and root branching (Ramasamy and Mahawar, 2023). Also, the introduction of microbes changing plant stress responses can make a synthetic antibiome resistant to both heat and flooding. For example, some anaerobic bacteria will be working to keep the transport of nutrients going during long floods, while heat tolerant endophytes will be taking care of the plant’s antioxidant overhaul via enzyme systems (Ajuna et al., 2024). Apart from this, the planned establishment of these microbial ecosystems will be a green replacement for chemical fertilizers and anti-stress products as the plants’ resistance is raised (Kaiser et al., 2023; Lu et al., 2025; Tamrakar et al., 2025). As a result, the use of synthetic microbial populations is a pioneering approach to the production of climate-resilient crops and a decrease in the agricultural sector’s need for these environmentally harmful inputs (Basu et al., 2021; Ramasamy and Mahawar, 2023; El-Saadony et al., 2024).

Deciding whether to actively engineer microbial communities or rely on natural selection depends on the degree of environmental constraint, desired function, and complexity of the target traits (El-Saadony et al., 2024; Roghair Stroud et al., 2024; Ma J. et al., 2025). In systems where natural microbial diversity already provides the required functions such as disease suppression in historically healthy soils, nutrient cycling in fertile sites, or stress mitigation in moderately challenging environments selective enrichment or top-down approaches may suffice. These strategies leverage existing ecological interactions, require less manipulation, and typically exhibit higher environmental stability (Basu et al., 2021). In contrast, engineering is often necessary when the desired functional traits are rare, multi-faceted, or poorly represented in native communities, such as introducing novel metabolite pathways, multi-stress resilience, or highly specific plant-growth promotion in degraded or marginal soils. Bottom-up design or adaptive evolution approaches allow precise incorporation of complementary traits, functional redundancy, and stress tolerance, but at the cost of increased time, effort, and regulatory oversight. Ultimately, the choice between harnessing nature and active engineering should be guided by trait availability, ecological context, and risk–benefit considerations, aiming for interventions that maximize functionality while minimizing ecological disruption (Ramasamy and Mahawar, 2023).

The geographic transferability of microbial inoculants or engineered communities their ability to function effectively across different soils, climates, and cropping systems is a major challenge in rhizosphere bioengineering (El-Saadony et al., 2024). Microbial performance is strongly context-dependent, influenced by local soil physicochemical properties (pH, texture, organic matter), climate factors (temperature, precipitation), native microbial community composition, and host genotype. Studies show that microbial consortia performing well in one location often fail to establish, persist, or confer intended benefits when transferred to a geographically distinct environment (Basu et al., 2021; Ramasamy and Mahawar, 2023; Ajuna et al., 2024). For example, arbuscular mycorrhizal fungi or P-solubilizing bacteria adapted to temperate soils may exhibit reduced colonization or functionality in tropical or arid systems. Even top-down approaches relying on enriched natural communities may face limited transferability if native microbial interactions or keystone taxa are absent in the new environment (Ajuna et al., 2024).

Strategies to improve geographic transferability include selecting broadly adapted, stress-tolerant strains, designing consortia with functional redundancy, and pre-conditioning microbes under multiple environmental conditions prior to deployment (Das et al., 2025). Nonetheless, predictive frameworks for cross-site functionality remain underdeveloped, and extensive field testing across diverse sites is often necessary before widespread application. Native soil and rhizosphere microbes often exhibit a “home-field advantage,” whereby locally adapted strains establish more efficiently, persist longer, and maintain functionality better than introduced or engineered consortia. This advantage arises from long-term co-evolution with the local soil environment, native plant hosts, and existing microbial networks (Karnwal et al., 2025; Mansoor et al., 2025). Factors contributing to home-field advantage include optimized nutrient acquisition under local soil chemistry, competitive exclusion of non-native taxa, and resilience to site-specific abiotic stresses such as temperature extremes, moisture variability, or salinity. Empirical studies show that even high-performing inoculants can be outcompeted by native microbiota, leading to reduced colonization and functional impact when deployed outside their native environment (Ferrocino et al., 2023). This phenomenon has important implications for microbiome interventions: (i) it highlights the value of selecting or enriching locally adapted strains rather than relying solely on imported inoculants; (ii) it suggests that synthetic communities designed for one site may require site-specific adaptation or pre-conditioning for success elsewhere; and (iii) it underscores the ecological principle that interventions must complement, rather than displace, existing microbial networks to achieve stable and effective outcomes.

### 5.3 Bioengineered rhizospheres for carbon sequestration and climate mitigation

With the help of microbial biotechnology breakthroughs, changing root systems in a manner that increases the plant’s resistance to stress but also leads to quick carbon uptake and climate change mitigation has become possible (Ajuna et al., 2024; Das et al., 2025). The area around the roots is the main broadcasting point for the carbon cycle because root exudates give carbon to microorganisms and, in turn, influence SOC accumulation. By using bioengineering techniques, the rhizosphere microbial communities can be altered or manipulated so as to increase carbon stabilization and, consequently, reduce greenhouse gas emissions (Das et al., 2025; Karnwal et al., 2025). One of the possible ways to go about this is by selecting or even genetically modifying microorganisms that promote the production of long-lasting soil organic molecules. These microorganisms producing lignin-like or polysaccharide-like extracellular substances might in the future be very effective in storing carbon by gluing live matter onto soil minerals. Creating microbial consortia that will enhance the rates of root exudation and thus soil carbon flow is another strategy. It would be possible, with the right kind of bioengineering, for certain kinds of bacteria such as some strains of Streptomyces and Rhizobium to convert the carbon dioxide from the root metabolism into more stable forms which would be stored underground for a long time (Karnwal et al., 2025). Besides, the precipitation of calcite and other microorganisms-induced formation of minerals, particularly of carbonated minerals, have enhanced the soil carbon sequestration through the microbial-produced process known as MICP. Furthermore, by manipulating the soil chemical processes, certain microbes can also minimize the release of potent greenhouse gases such as nitrous oxide and methane to the atmosphere. In essence, these engineered rhizospheres not only help in the main goal of mitigating climate change, which is through plant growth under stress conditions and acting as biological carbon sinks. This is one bold step toward a sustainable and climate-smart agricultural system (Mansoor et al., 2025).

Quantitative estimates of carbon sequestration vary widely depending on system and measurement scale. Under improved management practices such as cover cropping, no-till, and agroforestry, soil carbon gains are often in the range of 0.3–1.2 t C ha^–1^ yr^–1^ in agricultural soils, though these encompass plant and microbial contributions (Das et al., 2025). Microbial necromass has been shown to increase SOC by ∼9–42 % under certain management regimes, indicating a potentially large role for microbial processes in SOC formation. Direct microbial CO2 fixation rates measured in controlled soils span ∼4–18 mg C kg^–1^ soil d^–1^, but converting such rates to field-scale sequestration remains difficult (Karnwal et al., 2025). Importantly, robust quantitative data that isolate the specific contribution of engineered or naturally selected microbes to long-term soil carbon sequestration remain limited, representing a significant knowledge gap for translating mechanism to mitigation potential (Mansoor et al., 2025).

While soil carbon sequestration is a key strategy for mitigating climate change, it does not always directly correlate with enhanced crop productivity. Several factors contribute to this trade-off, particularly when management practices designed to increase soil carbon storage also alter the soil environment in ways that can affect crop growth (Kumar et al., 2022). For instance, practices like cover cropping, reduced tillage, and organic amendments can increase soil organic carbon (SOC) over time, but they may not always lead to immediate increases in crop yields. The additional organic matter in the soil can improve soil structure, water-holding capacity, and nutrient cycling, which may enhance productivity in the long term. However, in the short term, some practices, such as increased biomass retention, may tie up soil nutrients (especially nitrogen) in organic forms, leading to nutrient limitations for crops (Hennig et al., 2015; Kumar et al., 2022).

Another critical factor is that microbial-driven soil carbon sequestration while beneficial for carbon storage can sometimes lead to competition between soil microbes and plants for nutrients or carbon. Soil microbes that increase SOC through decomposition and stabilization processes may outcompete plant roots for available resources, especially nitrogen, leading to short-term yield reductions (Hennig et al., 2015; Kumar et al., 2022; Ferrocino et al., 2023). Additionally, if microbial processes that contribute to carbon sequestration (e.g., fungal mycorrhizal networks or microbial immobilization of nutrients) are not properly balanced, the result can be a temporary decrease in crop productivity, particularly in nitrogen-limited systems. On the other hand, when microbial communities are optimized to support plant growth and nutrient availability, their contribution to both carbon sequestration and crop productivity can be synergistic (Kumar et al., 2022). Ultimately, the balance between carbon storage and crop productivity depends on management practices, such as the type and quantity of organic inputs, crop rotation, and the microbial species selected or encouraged for the rhizosphere. In some cases, optimizing microbial communities for carbon sequestration may involve a trade-off with immediate crop yields, but over time, improved soil structure, enhanced water retention, and long-term nutrient cycling can lead to greater resilience and sustained productivity. Thus, integrated management approaches that consider both carbon sequestration and crop productivity are necessary to achieve the dual goals of agricultural sustainability and climate mitigation (Kumar et al., 2022). Soil carbon sequestration is inherently limited by the concept of carbon saturation, which posits that soils have a finite capacity to stabilize organic carbon in mineral-associated pools. Once this capacity is approached, additional inputs of organic matter or microbial carbon may no longer result in significant long-term sequestration. This is particularly relevant for agricultural soils that already have moderate to high baseline soil organic carbon (SOC) levels. In such soils, even with intensive management practices such as cover cropping, reduced tillage, or organic amendments, the incremental gains in stabilized SOC may be minimal (Hennig et al., 2015).

### 5.4 Case studies in major crops (rice, wheat, maize, legumes)

Several case studies on major agricultural commodities, including wheat, maize, rice, and legumes, pinpoint the importance of intra-plant and microbe interactions for efficiency under climate stress (Ferrocino et al., 2023; Karnwal et al., 2025; Mansoor et al., 2025). In rice-a crop subjected to periodic flooding-some bacterial associations have been found to mitigate methane emission, coupled with betterment of plant health. Methanotrophic microbial species residing within the rhizosphere enhance nutrient availability and reduce greenhouse gas emission by oxidizing carbon dioxide emitted by methanogens. Besides, rice root structure and resilience under submerged conditions have been reported to be enhanced with inoculation of *Azospirillum* and *Pseudomonas* strains (Kumar et al., 2022; Ferrocino et al., 2023).

Among the major yield-affecting factors in wheat, heat and drought have a key position. Inoculation of the bacterium *amyloliquefaciens* together with bacteria *Arthrobacter* species enhance drought tolerance by increasing root length, chlorophyll content, and activities of antioxidant enzymes (Kumar et al., 2022). In the same vein, wheat plants colonized with *Trichoderma harzianum* display a higher sensitivity to temperature stress caused by increased nutrient uptake and modulation in genes responsible for stress response (Hennig et al., 2015).

Maize is one of the most important agricultural products in the world and faces severe threats from drought and nutrient depletion. Inoculation of microbial consortia comprising bacterial species *Azospirillum brasilense*, *Pseudomonas putida*, and *Enterobacter cloacae* has significantly enhanced the drought endurance in plants through the induction of root development and hormone regulation (Atkinson et al., 2022; Atkinson et al., 2022). The beneficial microbes also promote phosphorous solubilization and nitrogen fixation to maintain continuous nutrient supply under stress conditions (Atkinson et al., 2022).

**FIGURE 4:**
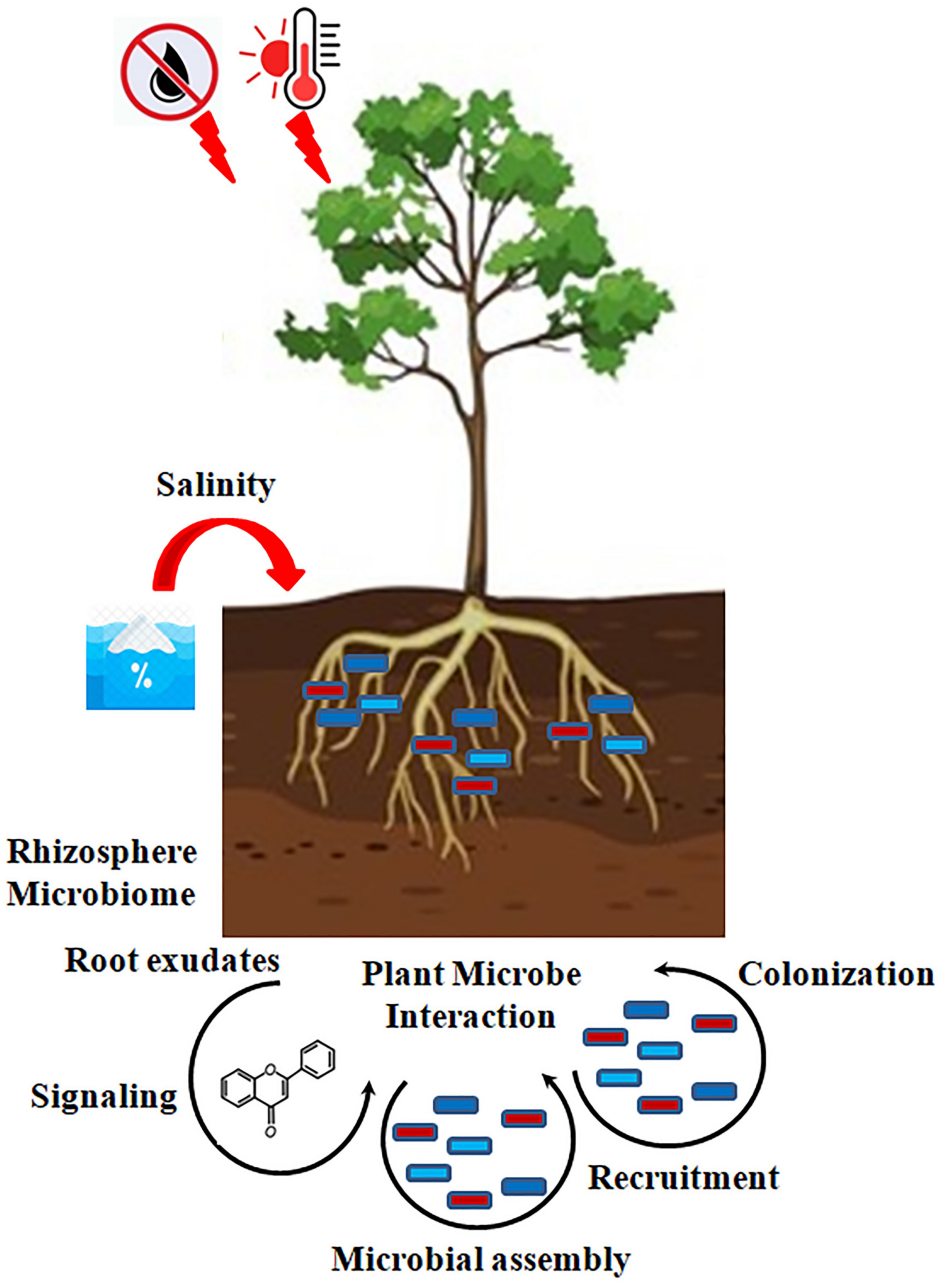
Schematic overview of plant responses to abiotic stress and their interactions with the rhizosphere. Heat- and water-deficit stresses aboveground affect plant physiological status, while reduced soil water influences root functioning. In the rhizosphere, roots interact with diverse microbial communities. Stress conditions impact the composition of rhizosphere microbiota, which in turn affects its activity. Phenolic/flavonoid compounds are among major plant secondary metabolites released via roots into the rhizosphere. They represent important players in microbe-root communication and influence nutrient intake, plant stress response, and resilience.

For legumes, whose productivity is heavily dependent on that relationship, it remains key to maintaining an effective mutualistic relationship via nitrogen-fixing spores under salt water and heat stress (Atkinson et al., 2022). The inoculation with salt-resistant bacteria strains has improved the nodulation efficiency and rates of nitrogen uptake in saline-grown chickpeas and soybeans. Spraying plants with mycorrhizal mushroom arbuscular mushrooms (AMF) also enhances crop-water relations and boosts phosphorus uptake during dry spells. Together, these examples illustrate how manipulation of plant-microbe interactions can maintain crop productivity and soil health in a changing climate (Figure 4; McCarty and Ledesma-Amaro, 2019).

The complex interactions between microorganisms and rhizosphere microbiota present the basis for ecosystem stability and plant durability. Severe climatic hazards in the form of heat, rainfall, drought, and salinity further threaten these very relationships and global food security (Yu et al., 2022). However, emerging strategies in synthetic microbiomes and bioengineered rhizospheres have pointed to ways forward on such issues. Utilizing microbial diversity through the creation of targeted microbial consortia strengthens the plant defense against climate extremes and contributes to climate change mitigation. Case studies related to grains, cereals, rice, maize, and beans outline the benefits of such approaches. There is an immediate need to incorporate microbial approaches into climate-smart agriculture to ensure environmental resilience and sustainable agriculture under global weather uncertainties (Kumar, 2023).

## 6 Tools and technologies enabling synthetic microbiome research

Research into engineered rhizospheres and synthetic microbiomes has provided novel avenues for enhancing plant resilience against climate stressors such as salinity, drought, and nutrient deficiencies (Kumar, 2023). Synthetic microbiomes, in contrast to conventional microbial inoculants, are logically constructed groups of advantageous microorganisms put together to replicate or enhance natural ecosystem functions throughout the rhizosphere (Wu L. et al., 2024).

### 6.1 Multi-omics (metagenomics, metatranscriptomics, metabolomics)

The precise connecting of diversity of microbes, metabolic processes, and network functions within plant-associated microbiota has been made possible by recent developments in multi-omics tools such as genome sequencing, metatranscriptomics, metabolomics, and proteomics (Alzate Zuluaga et al., 2024; Nunes et al., 2024; Blonde et al., 2025). These discoveries enable scientists to pinpoint the keystone species and core microbial taxa that promote development in plants and stress tolerance. A thorough understanding of the composition of microbial communities and the potential for functional genes is offered by metagenomics (Alzate Zuluaga et al., 2024). Researchers can discover keystone categories and metabolic capacities essential for nutrient cycling, resilience to stress, and development of plants promotion by analyzing total DNA about rhizosphere samples. This is complemented by metatranscriptomics, which identifies the genes that have been actively expressed by bacterial populations under specific environmental or plant-induced conditions. In such a way, it would be easier to understand dynamic interactions, such as how microbes respond to drought, salinity, or root exudates. This approach now captures the chemical fingerprint of the interactions between plants and microbes by profiling small molecules such as phytohormones, their additional products from metabolism, and signaling compounds. Such insight will be key to linking microbial activity to plant phenotypic outcomes (Sahoo et al., 2025).

High-throughput microbiome research and rhizosphere engineering rely on complex multi-omics datasets, including 16S/ITS amplicon sequencing, metagenomics, metatranscriptomics, and metabolomics (Nunes et al., 2024). Processing these datasets requires robust data analysis pipelines for quality control, taxonomic/functional assignment, network inference, and statistical modeling (Sahoo et al., 2025). Commonly used pipelines include QIIME2, DADA2, mothur for amplicon analysis, and MetaPhlAn, HUMAnN, or MG-RAST for metagenomics. Downstream integration often involves machine learning, co-occurrence network analysis, and multi-omics data fusion, which demand substantial computational resources, including high-memory servers or cloud-based high-performance computing (Tariq et al., 2025). Proper experimental design, standardized pipelines, and adequate computational infrastructure are essential to ensure reproducibility, minimize bias, and extract actionable insights for microbiome engineering (Ullah et al., 2025).

### 6.2 Synthetic biology platforms for microbial design

Synthetic biologists redesign life in a systematic way, using principles of engineering like modular DNA “parts” and computer simulations. Recent developments in genetic engineering, and especially CRISPR/Cas systems, have made the exact insertion, deletion, or regulation of genes easier (Tariq et al., 2025). The development of fast DNA assembly methods allows to build metabolic pathways and biological circuits able to precisely control cellular behavior. Synthetic biology, as it fundamentally relies on DNA assembly and genetic manipulation, provides a set of tools that are particularly useful for the control of microbes within microbial consortia (Ullah et al., 2025). These include the use of exogenous molecules to control population behavior, construction of interdependent signaling systems to enable communication among microbes, and establishment of interdependent networks of bacteria via syntrophic interactions. Such approaches can be integrated to enable precise design, assembly, and control of collaborative microbial communities (Kumar et al., 2024).

### 6.3 Intercellular signaling to coordinate microbial communication

Artificial communities of microbes can be generated by utilizing natural biological communication mechanisms, including biological messaging, physical interaction, molecule attachment, ion channel channels, and electrical wiring (Ullah et al., 2025). The best described example is the quorum sensing QS phenomenon, which involves the release of small molecules (autoinducers) by microorganisms. When the population grows, so does the concentration of the concerned molecules, which allows cells to perceive their level of concentration and vary gene expression accordingly. This process allows microbes to determine when to undertake unified interpersonal actions (Tan et al., 2022; Kumar et al., 2024; Ullah et al., 2025).

### 6.4 Engineered syntrophies to build codependent strains

Most natural microbial communities display metabolic interdependencies and cross-feeding interactions. By drawing inspiration from these interactions, synthetic consortia can be designed to enter into syntrophic relationships, where one life form relies on metabolic byproducts produced by another. Commonly, these consortia are constructed with co-auxotrophic strains where each member is lacking a required metabolite and cannot survive unless another member produces it. This intentional codependency stabilizes the community and promotes cooperation among the members of the consortium (Patyal et al., 2025).

### 6.5 Synthetic biology tools for building microbial consortia with defined behaviors

The synthetic biology tools used to construct individual organisms are equally applicable to designing complex functions in microbial consortia. Syntrophic relationships, outside stimuli, and intercellular signaling techniques can be combined to influence the behaviors of such microbial communities. In addition, these techniques allow division or cooperation of labor among community members, modification of population sizes, and control over the spatial distribution of microbes within a consortium (Tan et al., 2022).

### 6.6 Computational modeling of microbial networks

Computational modeling has emerged as a potent technique for analyzing, predicting, and controlling complex interactions within microbial communities and synthetic microbiomes. Microbial communities, including both natural and synthetic ones, exhibit complex interactions of competition and cooperation as well as quorum sensing and metabolite exchange under various environmental limitations, making their interactions highly interconnected and difficult to analyze directly (Patyal et al., 2025). For instance, genome-scale metabolic models (GEMs) were used to infer metabolic cross-feeding relationships among the members of synthetic consortia, with the model predictions successfully informing the design of stable consortia for nitrogen fixation and phosphorus solubilization. Meanwhile, dynamic flux balance analysis (dFBA) offers the power to simulate time-dependent effects of metabolite exchange, facilitating the study of the effects of changing carbon supply or the presence of oxygen on consortium stability. Furthermore, agent-based models were used to investigate the effects of the spatiotemporal organization of the rhizosphere microbial community, with the model successfully illustrating the influence of root exudation on microbial colonization (Shade, 2018; Tan et al., 2022; Patyal et al., 2025).

Network-based strategies such as microbial interaction networks and co-occurrence modeling have been employed to identify a set of key taxa that contribute to the major functions of microbiomes and their fragility under stressful environments such as drought or nutrient starvation. These computational tools help to close gaps between experimental microbiome engineering models and applications by enabling hypothesis testing and prediction of management strategies (Tan et al., 2022).

### 6.7 Bioreactors and lab-on-chip systems for rhizosphere simulation

MBRs were originally developed at the interface of microfluidic technology and molecular biology. Their origin goes back to the early 1950s, when improvements in photolithography made the techniques for microfabrication possible and the first miniature devices were realized, including micro-sized transistors (Patyal et al., 2025). One of the new uses for the microtechniques developed involves the much smaller size of chemical and biological testing. This development has allowed researchers to derive specific and high-resolution information using minimal sample amounts, and it thus enabled the design of modern bioreactors that incorporate effectiveness, precision, and flexibility in experimental format (Shade, 2018; Tan et al., 2022; Patyal et al., 2025). Plant-micro interactions within complex natural soils are challenging to study due to the heterogeneity and dynamic nature of the rhizosphere. To precisely study the action of microbes, variation in nutrients, and response of plants, biological reactors and lab-on-chip systems offer very controlled conditions closely resembling those of the rhizosphere. Bioreactors provide scalable platforms that enable accurate regulation of temperature, humidity, oxygen, and nutrient delivery. They enable researchers to study how microbial communities respond to environmental stress, nutrient availability, and the introduction of modified strains. Lab-on-a-chip systems minimize the root infrastructure into microfluidics-based instruments, thus allowing for the observation and tracking of colonization by microorganisms and the development of roots and chemical gradients in real time. LoCs are compact, portable implements that allow precise control and measurement of processes in biology. Because they facilitate the collection of samples and transportation, extraction, and analysis, they are particularly helpful in the management of chronic diseases or outbreaks of an epidemic. Such LoC systems greatly enhance illness coverage and surveillance at the population level by facilitating high-throughput testing and analysis. The study of relationships between populations of plants and microbes depends upon the distribution of root exudates and delivery of nutrients and spatial heterogeneity, which can be emulated using these systems. Integration with detectors, photography, and omics technologies allows for the assessment of bacterial activity, communication via metabolites, and dynamics of communities with high resolution. Using bioreactors and lab-on-a-chip platforms in the recreation and manipulation of the rhizosphere, the investigator can create synthetic microbial communities and biologically generated microbial communities for agriculture that is resilient to climate within a much shorter period than usual (Boyle et al., 2024).

Translating rhizosphere microbiome research from the lab to the field requires scalable platforms for testing, optimizing, and producing microbial consortia. Bioreactors allow controlled cultivation of complex microbial communities under defined environmental conditions, enabling precise control over nutrient availability, oxygen levels, pH, and temperature (Patyal et al., 2025). They are particularly useful for studying community dynamics, testing stress responses, and producing microbial inoculants at a scale sufficient for greenhouse or pilot field trials. On the other hand, lab-on-chip systems provide microfluidic platforms that simulate rhizosphere microenvironments at high throughput, enabling real-time monitoring of microbial interactions, signaling, and functional outputs under varying soil or plant conditions. These systems facilitate rapid screening of strains or consortia before field deployment. While both platforms offer valuable scalability advantages, each has limitations (Shade, 2018). Bioreactors provide larger-scale biomass production but may not fully replicate soil microenvironments, whereas lab-on-chip devices allow precise mechanistic studies but are limited in volume and complexity. Integrating insights from these platforms can guide rational design of microbial consortia, pre-select stress-tolerant strains, and optimize inoculant formulations for field-scale application. Ultimately, combining lab-on-chip screening with bioreactor production forms a scalable pipeline bridging lab discovery and real-world agricultural deployment. Soil is inherently heterogeneous, both spatially and temporally, in terms of physical structure, chemical composition, moisture distribution, and native microbial communities. This heterogeneity poses significant challenges for microbiome engineering, as introduced or engineered consortia may not establish uniformly across different microenvironments. For instance, variations in pH, organic matter content, nutrient availability, and soil texture can lead to uneven microbial colonization, altered metabolic activity, and variable functional outcomes (Boyle et al., 2024). Patchiness in moisture or aeration can further influence microbial growth and signaling, affecting the consistency of stress mitigation, nutrient cycling, or carbon sequestration. Even within a single field, microbial inoculants may perform well in some soil microhabitats while failing in others, limiting overall effectiveness. Addressing soil heterogeneity requires strategies such as site-specific inoculant formulation, functional redundancy in microbial consortia, or precision application methods. High-throughput screening in lab-on-chip systems or microcosms that mimic heterogeneous soil environments can help identify strains that tolerate variability, while bioreactor-based pre-conditioning can enhance microbial resilience. Incorporating soil heterogeneity into predictive models is essential for realistic assessment of microbiome interventions at the field scale (Singh and Rastogi, 2024).

### 6.8 AI and machine learning in predicting plant–microbe interactions

**FIGURE 5:**
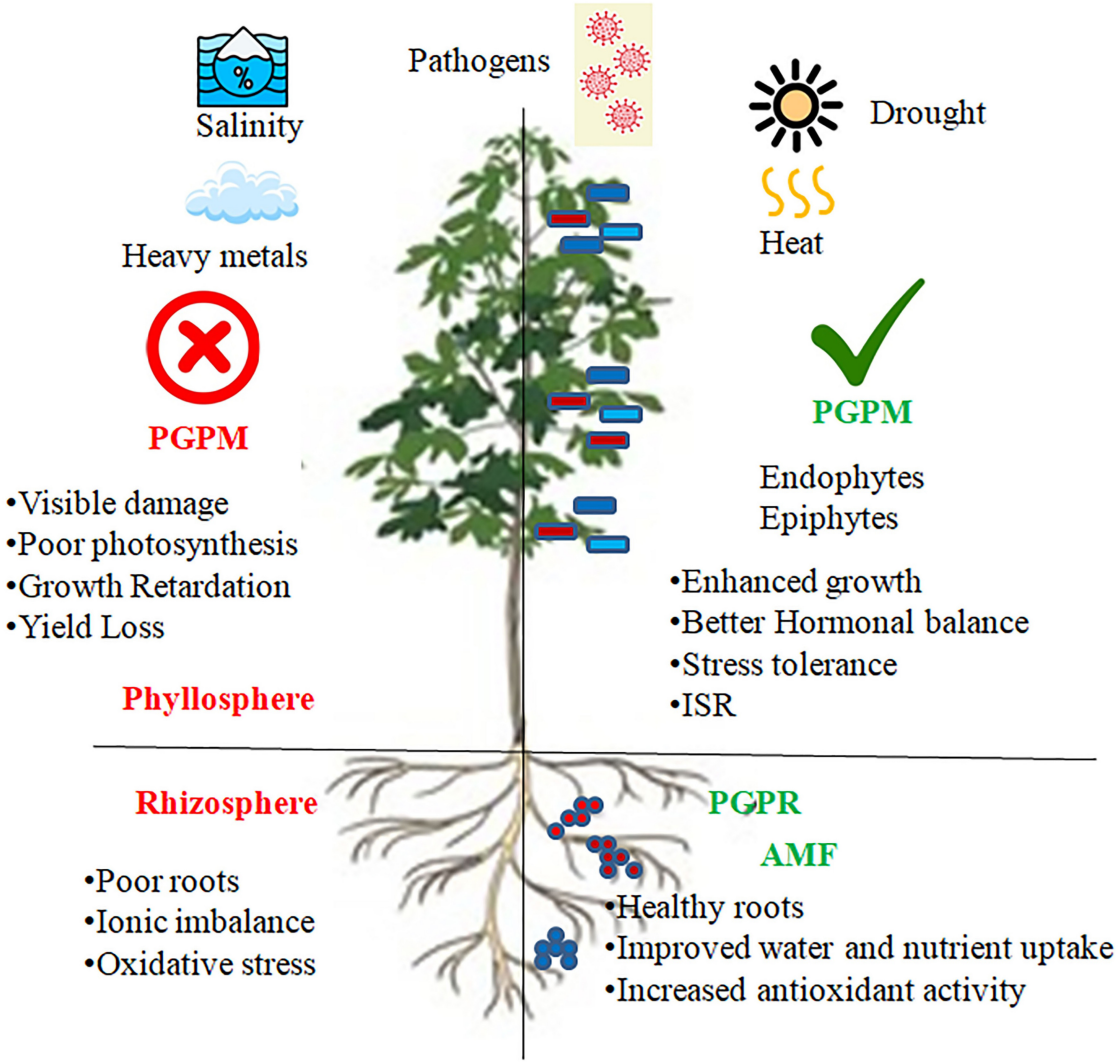
Workflow of data-driven design and validation of synthetic microbial communities. This figure provides a three-tier framework that describes compositional and functional characterization of microbial communities, data-driven hypothesis generation through statistical and network analyses, and experimental validation in *in vitro* and *in vivo* models. This iterative approach connecting community structure with function will guide and enable rational design of synthetic microbiomes.

Plants live in a noisy microbial neighborhood, and AI is becoming the megaphone that helps us hear which voices matter (Table 4). Recent work shows that machine-learning models can predict whether an introduced microbe will successfully colonize a community and even estimate its steady-state abundance from baseline community data, turning previously unpredictable invasion outcomes into actionable forecasts (Singh and Rastogi, 2024). That predictive power is already being applied to synthetic microbiomes (SynComs) and bioengineered rhizospheres: researchers combine reductionist SynCom experiments with ML to identify community patterns and taxa that confer disease protection, nutrient mobilization, or stress tolerance a fast route from hypothesis to candidate consortia. Beyond pattern recognition, the field is moving toward mechanistic integration: multi-omics (metagenomes, transcriptomes, metabolomes) and genome-scale metabolic models are being fused with ML and deep learning so models can both predict outcomes and suggest causal mechanisms (for example, which root-exudate catabolic pathways let a microbe thrive in a rhizosphere) (Figure 5 and Table 5). This makes it possible to rationally design SynComs or edit plant exudation to favor beneficial partners (Parker and Kunjapur, 2020). Taken together, these advances offer a practical roadmap to climate-resilient agriculture: AI speeds discovery (finding the few keystone strains in a sea of microbes), helps predict field performance, and guides bioengineering of plant–microbe communication so crops better withstand drought, heat, and disease. Challenges remain—data standardization, ecological transferability from lab to field, and ethical/regulatory questions—but the combination of SynCom experiments, metabolic models, and ML is already reshaping how we engineer rhizospheres for resilient food systems (Parker and Kunjapur, 2020; Singh and Rastogi, 2024).

**TABLE 4:** Comparison of common AI/ML approaches in rhizosphere microbiome study.

Tool/approach	Strengths	Limitations	Best use case
Linear regression/logistic regression	Simple, interpretable, fast	Limited ability to model non-linear relationships, poor for complex community interactions	Predicting simple relationships between microbial abundance and plant traits
Decision trees/random forest	Handles non-linear relationships, robust to noise, feature importance ranking	Can overfit small datasets; less interpretable than linear models	Feature selection, medium-complexity datasets, identifying key taxa
Gradient boosting (XGBoost, LightGBM)	High predictive accuracy, handles heterogeneous data	Requires careful hyperparameter tuning; can be computationally intensive; black-box	Predicting functional outcomes, complex microbial interactions
Neural networks/deep learning	Captures highly complex, non-linear interactions; integrates multi-omics and environmental data	Black-box, needs large datasets, high computational cost, difficult to interpret	Multi-omics integration, large-scale prediction of consortia performance
Explainable AI (SHAP, LIME)	Adds interpretability to black-box models; identifies influential features	Additional computation; may not capture all interactions perfectly	Mechanistic insight from complex models, guiding experimental validation

**TABLE 5:** Tools and technologies for bioengineering rhizospheres and synthetic microbiomes.

Technology/tool	Function/principle	Application in synthetic microbiomes or rhizospheres	Limitations	References
Metagenomics	Studies genetic material of all microbes in a sample	Identifies microbes present and their potential functions	Needs bioinformatics; may miss rare species	(Handelsman, 2004; Quince et al., 2017)
Metabolomics	Studies chemical/metabolic products of microbes and roots	Understands root–microbe chemical communication	Complex analysis; metabolites degrade quickly	(Fiehn, 2002; van Dam and Bouwmeester, 2016)
Metatranscriptomics	Analyzes actively expressed microbial genes under specific conditions to reveal functional activity	Reveals microbial responses to plant or environmental signals.	High quality RNA, limited temporal insight, sensitive to degradation;	(Moran et al., 2013; Shakya et al., 2019)
Microfluids (lab-on-chip systems)	Simulates microenvironments, enables high-resolution microscale imaging	Enables *in situ* analysis with high spatio-temporal resolution.	Limited device designs; soft lithography is costly and slow, hindering rapid prototyping.	(Whitesides, 2006; Stanley et al., 2016)
CRISPR and synthetic biology	Edits or designs microbial genes	Produces engineered microbes for plant growth, stress tolerance, or disease control	Safety and regulatory concerns; ecological risks	(Jinek et al., 2012; Ke et al., 2021)
Computational modeling and AI	Uses computer models to predict microbe interactions	Helps design stable and compatible synthetic microbial consortia	Inadequate data quantity and quality, Needs large datasets; may not work in real soil	(Widder et al., 2016; Ryo and Rillig, 2017)

Artificial intelligence (AI) and machine learning (ML) are increasingly being applied to analyze complex rhizosphere microbiome datasets, predict microbial interactions, and optimize microbial consortia for plant growth and stress resilience. These approaches are particularly useful for integrating multi-omics data (metagenomics, metatranscriptomics, metabolomics) and environmental parameters to identify key taxa, functional modules, or predictive markers of plant performance (Singh and Rastogi, 2024).

The performance of AI and machine learning models in rhizosphere microbiome research critically depends on the quantity, quality, and diversity of training data. High-quality datasets should include well-annotated microbial community profiles (16S/ITS/metagenomics), functional traits, environmental metadata (soil type, pH, nutrient status, moisture), and host plant characteristics. Insufficient or biased training data can lead to overfitting, spurious correlations, and poor predictive performance when models are applied to new environments. Diverse and representative datasets are particularly important to capture the inherent spatial and temporal heterogeneity of soils, the variability of microbial interactions, and the context-dependent nature of plant–microbe relationships. Moreover, standardized data preprocessing, normalization, and curation are essential to minimize technical noise and batch effects, which can otherwise propagate errors into downstream predictions. Ultimately, robust AI/ML applications require large-scale, high-quality, multi-site, and multi-omics datasets, combined with careful validation and cross-site testing to ensure models are generalizable and actionable for microbiome engineering (Singh and Rastogi, 2024).

A key challenge in applying AI and machine learning to rhizosphere microbiomes is the trade-off between model interpretability and predictive performance. Simple models, such as linear regression or decision trees, are highly interpretable: researchers can readily understand which microbial taxa, environmental variables, or functional traits drive predictions. However, these models may lack the capacity to capture complex, non-linear interactions among microbial communities, plant hosts, and environmental factors. In contrast, “black-box” models, such as deep neural networks or ensemble methods (e.g., Random Forest, XGBoost), often achieve higher predictive accuracy by capturing these intricate relationships but at the cost of transparency. The lack of interpretability can hinder mechanistic understanding, limit confidence in the biological plausibility of predictions, and complicate regulatory or field deployment decisions (Parker and Kunjapur, 2020).

Emerging solutions include explainable AI (XAI) approaches, such as SHAP (Shapley Additive Explanations) values, LIME (Local Interpretable Model-agnostic Explanations), and feature importance rankings, which can provide insight into how black-box models make predictions. By combining high-accuracy models with interpretability tools, researchers can balance predictive performance with mechanistic insight, enabling more informed design of microbial consortia, selection of functional traits, and deployment strategies in variable soil and climatic conditions. Nevertheless, achieving a full mechanistic understanding from black-box models remains challenging, and experimental validation remains essential (Parker and Kunjapur, 2020). While AI and machine learning have great potential for rhizosphere microbiome analysis and consortia design, not all tools are equally effective across tasks, and each has inherent limitations. Selecting the appropriate model depends on the research question, data type, and required balance between predictive power and interpretability.

## 7 Applications in climate-resilient agriculture

Climate change is intensifying environmental stresses including drought, heat, salinity, flooding, nutrient limitation, and unpredictable precipitation (IPCC, 2022). Traditional agricultural practices are increasingly inadequate (Trivedi et al., 2020). Harnessing plant-microbe interactions through synthetic microbial communities (SynComs), engineered rhizospheres, and bioinoculants is emerging as a promising frontier to build climate resilience (Table 6). Advances in metagenomics, synthetic biology, and computational modeling now permit us to design and deploy microbiomes with targeted functions (Widder et al., 2016; Ke et al., 2021).

**TABLE 6:** Applications of synthetic microbiomes and bioengineered rhizospheres in climate-resilient agriculture.

Application area	Mechanism of action	Primary climate stress	Outcome	Key microbial strains/ consortia	Research insight	References
Drought tolerance	Engineered microbes enhance root growth and osmolyte accumulation	Drought	Improved water-use efficiency and plant survival under water stress	*Bacillus amyloliquefaciens, Bacillus subtilis*, synthetic consortia	Synthetic *Bacillus* consortia shown to promote drought resilience in cereals	(Kasim et al., 2013; Timmusk et al., 2014)
Nutrient use efficiency	Bioengineered rhizobacteria fix nitrogen and solubilize phosphorus more efficiently	Nutrient-depleted soils	Reduced dependence on chemical fertilizers and improved soil fertility	*Rhizobium, Azotobacter, Pseudomonas*	Synthetic microbial consortia designed for maize and rice nutrient optimization	(Curatti and Rubio, 2014; Herrera Paredes et al., 2018)
Salt stress mitigation	Halotolerant synthetic microbes regulate ion balance and stress-responsive genes	High soil salinity	Enhanced tolerance to salinity and improved yield in saline soils	*Pseudomonas putida, Flavobacterium, Alcaligenes, Halomonas*,	Engineered *Pseudomonas* strains improve salt tolerance in tomato and wheat	(Etesami and Beattie, 2018; Liu et al., 2025)
Disease suppression	Engineered microbes produce antimicrobial peptides and siderophores	Climate-induced pathogen proliferation	Reduced pathogen load and decreased pesticide usage	*Trichoderma, Bacillus velezensis*,	Synthetic microbiomes controlling *Fusarium* wilt and root rot in legumes	(Harman et al., 2004; Sharma et al., 2025)
Carbon sequestration	Microbes engineered to enhance root exudation and soil organic carbon accumulation	Climate change and rising CO2 levels	Improved soil carbon storage and mitigation of greenhouse gas emissions	*Streptomyces, Rhodococcus, and Pseudomonas*	Synthetic rhizosphere communities linked with enhanced carbon fixation	(Singh et al., 2010; Kuzyakov and Xu, 2013; Delgado-Baquerizo et al., 2016)
Heavy metal detoxification	Bioengineered strains induce heat-shock proteins and antioxidant enzymes	Heavy metal toxicity	Better adaptation to high-temperature environments	*Pseudomonas aeruginosa, Burkholderia cepacia, Enterobacter*	Synthetic endophytes improving thermotolerance in soybean and rice	(Grover et al., 2011; Ma et al., 2011; Vurukonda et al., 2016)
Integration with smart farming	Coupling microbial inoculants with IoT, AI, and sensor-based monitoring	Climate variability impacting soil and crop health	Real-time optimization of microbial performance under changing field conditions	AI-guided microbial consortia, smart biofertilizer cartridges	Precision agriculture platforms managing bioinoculant delivery and soil health tracking	(Mahlein, 2016; Wolfert et al., 2017; Liakos et al., 2018)

While biofertilizers and biostimulants offer significant potential for enhancing soil health, crop productivity, and sustainability, their widespread adoption in agriculture faces several barriers, many of which are tied to farmer decision-making. Understanding these barriers is the key to successfully scaling microbiome-based solutions in real-world farming systems (Table 7).

**TABLE 7:** Key points summary on different barriers, and its impact on adoption and mitigation strategy.

Barrier	Impact on adoption	Mitigation strategy
Economic considerations	High initial cost, low short-term ROI	Financial incentives, subsidies, improved cost-benefit evidence
Knowledge gaps	Uncertainty about effectiveness and application	Extension services, demonstration projects, education programs
Regulatory issues	Lack of standardization and certification	Clear regulatory frameworks and product certification
Field performance variability	Inconsistent results across soils, crops, climates	Customized formulations, on-farm trials, soil testing
Consumer demand	Low consumer awareness of bio-based products	Market incentives, consumer education campaigns, premium pricing
Long-term vs. short-term needs	Preference for immediate yield over sustainability	Highlighting long-term soil health and sustainability benefits

### 7.1 Biofertilizers and biostimulants based on synthetic consortia

Synthetic microbial consortia (SynComs) are carefully designed communities of beneficial microbes such as plant growth-promoting rhizobacteria (PGPR) and fungi assembled to act synergistically in enhancing plant growth and stress resilience (Herrera Paredes et al., 2018). Unlike single-strain inoculants, SynCom-based biofertilizers and biostimulants combine multiple functions including nitrogen fixation, phosphorus solubilization, phytohormone production, and induction of systemic resistance (Ke et al., 2021). According to recent research, these microbial combinations enhance root growth, nutrient uptake, and resistance to abiotic stressors like heat, salinity, and drought. Exopolysaccharide-producing strains of bacteria and fungi, for instance, improve soil aggregates and water retention, while other bacterial-fungal consortia alter root exudates to attract helpful microbes during stressful situations (Vurukonda et al., 2016). However, crop genotype, native microbiota composition, and soil type all affect their performance, highlighting the necessity of context-specific designs (Ray et al., 2020; Trivedi et al., 2020).

It is now possible to rationally design stable syntheses with enhanced formulations for field use thanks to developments in omics, biochemical modeling, and microbial ecology (Widder et al., 2016). In order to turn these environmentally friendly innovations into useful instruments for climate-resilient agriculture, future research will concentrate on enhancing persistence, adaptability, safety for humans, and regulatory approval (Ke et al., 2021).

One of the major challenges for the widespread adoption of biofertilizers and biostimulants is ensuring their formulation stability under a variety of environmental conditions. Unlike chemical fertilizers or synthetic pesticides, biological products are often sensitive to temperature fluctuations, pH, moisture, and exposure to UV light, which can reduce their viability and effectiveness. For instance, certain strains of beneficial microbes (e.g., nitrogen fixers, phosphate solubilizers) are highly susceptible to desiccation, which can occur during storage or transport, leading to a loss of microbial viability before application. Proper application methods are crucial for maximizing the performance of biofertilizers and biostimulants. Since these products often rely on living organisms or complex chemical interactions, improper application can result in poor colonization, ineffective nutrient cycling, or suboptimal plant growth responses (Widder et al., 2016).

### 7.2 Biofertilizer and biostimulant market

The global biofertilizer and biostimulant market has grown steadily in response to increasing demand for sustainable agriculture, soil health improvement, and reduction in chemical fertilizer use. Market dynamics are influenced by factors such as regulatory support, technological innovations, crop type, and regional adoption patterns. Despite promising scientific advances, commercialization faces several challenges including formulation stability, consistent field performance, regulatory hurdles, and farmer adoption barriers (Ke et al., 2021).

### 7.3 Microbiome engineering for sustainable crop intensification

By reimagining crops as plant microbial holobionts rather than isolated organisms, microbiome engineering reduces reliance on synthetic fertilizers while increasing yield, resilience, and nutrient efficiency (Trivedi et al., 2020). According to recent reviews, in many situations, engineered or carefully chosen consortia are more effective than single strains at providing a variety of complementary services like nutrient mobilization, hormone signaling, stress protection, and pathogen suppression (Herrera Paredes et al., 2018). Practical developments employ metabolic modeling, high-throughput omics, and synthetic biology to build communities with expected purposes and stable interactions. These tools now allow researchers to identify functional redundancy, select keystone taxa, and model the behavior of microbes under field-relevant stressors such as heat, salinity, and drought (Widder et al., 2016). When collaborations match nutrients with crop genetic makeup and soil context, field and laboratory studies show promising increases in efficiency of nutrient utilization and yield, and enhanced resistance to abiotic stress though results vary (Table 8). Contextually sensitive design and multiple-location trials are crucial because native soil ecosystems, environmental changes, and formulation/delivery limitations frequently limit persistence and reproducible results (Ke et al., 2021).

**TABLE 8:** Difference between microbiome engineering for intensification and biofertilizers and biostimulants.

Different features	Microbiome engineering for intensification	Biofertilizers and biostimulants
Focus	Engineering and optimizing microbial communities for specific functions	Commercialized products for plant growth and soil health
Stage	Primarily research and development (emerging technologies)	Market-ready products (commercialized)
Technology	Cutting-edge, including synthetic biology, genetic engineering, etc.	Commercial products, often naturally occurring microbes or compounds
Customization	Highly tailored microbial consortia designed for specific conditions	Typically more generalized, with limited customization
Application	Experimental, aiming for scalability in agriculture	Ready for field use, established application methods
Regulatory complexity	Potentially high (especially for GMOs or synthetic microbiomes)	Relatively lower (less stringent regulations for natural microbes)
Commercial viability	Not yet widely commercialized, still in R&D phase	Established, widely available in agricultural markets
Market focus	Advanced, research-driven solutions for agriculture intensification	Products targeting soil and plant health, already available to farmers

### 7.4 Integration with precision agriculture and smart farming technologies

New research illustrates how bioengineered rhizospheres and synthetic microbiomes increasingly complement smart farming systems and precision agriculture, opening up new avenues for climate-resilient and sustainable crop production (Trivedi et al., 2020). Precision agriculture involves data-driven technologies (such as detectors, drones, the IoT, and AI-based analytics) for real-time monitoring of the condition of the soil, nutrient fluctuation, and plant function. It is these technologies, in concert with engineered microbial consortia, which afford the unprecedented ability to precisely control the interactions between plants and bacteria, optimizing development-promoting functions with stress tolerance under changing climatic conditions (Wolfert et al., 2017). For instance, precision agriculture platforms can control the timing and placement of microbe-containing engineered inoculants, therefore maximizing productivity and minimizing resource losses. Such inoculants may be specifically formulated to match genotype and soil type. Smart sensors installed in the soil could enable feedback loops that inform adaptive management through monitoring microbial population growth, root transpiration rates, and nutrient bioavailability. AI models further reinforce this integration by offering forecasts around the responsiveness of microbial communities to changing environmental parameters like temperature, pH, and moisture (Ke et al., 2021). These combined innovations result in a synergistic paradigm wherein synthetic microbial communities play the role of dynamic bio-tools, while precision technologies represent management and surveillance systems. This integrated approach reduces the use of chemicals while enhancing crop yield along with the ability of such crops to respond to stressors like salt-water intrusion and protracted drought-a cornerstone of emerging global climate-smart and eco-friendly agricultural objectives (Liakos et al., 2018).

### 7.5 Role in reducing chemical fertilizer and pesticide dependence

This is the advancement in the area of plant protection and nutrition, where the advent of synthetic microbiomes and bioengineered rhizospheres are biological counterparts to traditional agrochemicals, thus enabling chemical fertilizers and pesticides to have their natural substitutes only in cases of emergencies (Trivedi et al., 2020). The presence of specific metabolites and signals allows the co-culture of bacteria that have been optimized for the above-mentioned functions to inhibit pathogens, enhance the uptake of nutrients, and support the development of plants. The removal of chemical fertilizers and the delivery of essential nutrients like potassium, phosphorus, and nitrogen instantly to plant roots are made possible by the deliveries of the mentioned artificial communities of bacteria, which are drastically reduced in terms of requiring chemicals that lead to soil degradation and water contamination (Herrera Paredes et al., 2018). Similarly, soil-borne pathogens are systematically resistant to bioengineered rhizospheres by generating lytic enzymes, siderophores, and antimicrobial compounds; furthermore, the drawing of pest populations in the soil at the same time is claimed as being a biological method of disinfestation. Besides that, the plant’s immune system is enhanced and the environment of harmful microorganisms is created through these systems so that the need for chemical pesticides is minimized (Ke et al., 2021). Additionally, the power of synthetic microbiomes to link up and sustain profitable microbial networks over time guarantees that even in harsh environments, such as with high salinity or drought, their performance remains steadfast, unlike the case with chemical inputs which often fail or cause greater environmental damage (Compant et al., 2010). The combination of these biological innovations with precision agriculture technologies greatly enhances their impact (Table 9). Administering and continually evaluating platforms can ensure that the microbial inoculants are optimized for timing and quantity, resulting in ecological balance and maximum efficiency. Thus, engineered root systems and synthetic microbiomes are not only able to carry out ecologically regenerative agricultural practices supporting global goals for the environment health and climate adaptation resilience but also to generate profitable production (Wolfert et al., 2017).

**TABLE 9:** The long-term sustainability concerns in terms of resilient agriculture.

Sustainability concern	Potential impact
Soil microbial diversity	Reduced native diversity, dependence on inoculants
Nutrient cycling	Imbalances, leaching, acidification
Carbon dynamics	Potential CO2 loss, reduced soil carbon storage
Ecological effects	Non-target species impact, horizontal gene transfer
Resistance development	Reduced effectiveness of biocontrol microbes
Economic/social	Dependence on proprietary products, farmer knowledge gaps
Resilience	Reduced adaptability under environmental variability
Regulatory/ethical	Long-term ecological uncertainty, GM concerns

## 8 Challenges and limitations

### 8.1 Ecological complexity and unpredictability of field environments

The large restraints in moving bioengineered rhizospheres and synthetic microbial communities from lab conditions to the field are an indication of their awesome power (Trivedi et al., 2020). Complexity and dynamics are intrinsic characteristics of natural ecosystems which make them subject to a variety of factors such as moisture, temperature, pH, and nutrient availability. These factors are unpredictable and can consequently lead to variability in the results of trials. The application of the artificially created microbial habitats and root systems in nature is still difficult because of the unpredictability of the natural ecosystems (Widder et al., 2016). There are also unpredicted conditions such as temperature, humidity, soil composition, and nutrient levels that can change the microbial life and activity. Furthermore, these factors may sometimes lead to inconsistent results when compared to lab experiments (Ke et al., 2021). Environmental stresses, soil mixing, and nutrient depletion from native communities as a result of dominating or interacting with introduced strains, are just some of the reasons why the effect of native microbes in the soil might be lessened. Moreover, the long-term ecological impact of genetically engineered microbes, including gene flow or extinction of native microorganisms, is still a subject of concern and lacks understanding. Future research needs to work on learning ecosystem interactions, leading to field stability and development of safe, adaptable methods for real-world Agricultural application to guarantee success (Compant et al., 2010).

### 8.2 Microbiome stability and persistence across soil types and climates

One of the major hurdles to the use of engineered microbial populations and bioengineered rhizospheres in different soils and climates is the difficulty in keeping them stable and persistent (Trivedi et al., 2020). A microbial community that is thriving in one place might become non-functional and unviable in another due to soil pH, nutrient arrangement, moisture, and temperature changes. These up-and-downs in the environment may affect the microbial interactions, the efficiency of colonization, and the expression of genes, leading to inconsistent results in the field (Ke et al., 2021). Moreover, the competition with the native soil microbes and changing seasonal patterns can lead to the collapse of the newly introduced microbial communities, thus impairing their efficacy over the long term. Hence, the need for resilient and adaptable microbial populations that can withstand and thrive in a variety of agroecological settings becomes imperative for consistent performance. The natural habitat of microbes, genome sequencing, and adaptive bioengineering will be the areas where further research is required for the synthetic microbiomes to achieve improved durability and resilience in the agricultural systems of the future (Widder et al., 2016).

### 8.3 Biosafety, regulatory, and ethical concerns in bioengineering

Synthetic microbiomes and bioengineered rhizospheres constitute a highly controversial issue in agriculture from the point of view of law, ethics, and biosafety. Among the concerns is the possibility of such ecological consequences, including among others, horizontal gene transmission, disruption of indigenous microbes’ populations, or non-targeted species being affected. These risks necessitate thorough biosafety assessments as a prerequisite for applying the technologies in large quantities in the fields (Ke et al., 2021). The laws regarding the use of genetically modified microorganisms vary significantly from country to country, and many are continuously changing to adapt to the progress in synthetic biology. In the absence of clear regulations, there is a likelihood of research translation and commercialization processes being slow. Besides, the issue of the developed biological assets ownership, environmental stewardship, and small-scale farmers’ socioeconomic impacts are some of the ethical concerns that are being raised (Compant et al., 2010). Transparent risk assessment, responsible innovation practices, and cooperation between countries to come up with biosafety regulations are the only way to overcome these business challenges. Engaging with scientists, policymakers, and the public will also contribute to ensuring that the biological engineering alternatives in crop cultivation are safe and socially accepted (Ricroch et al., 2011).

While microbiome engineering holds immense potential for agricultural intensification, these major challenges must be addressed to ensure that the technology can be successfully scaled in a sustainable and equitable manner. A holistic approach that combines technological, ecological, economic, and social considerations is essential for overcoming these barriers and ensuring that microbiome engineering contributes to long-term agricultural sustainability (Compant et al., 2010; Ricroch et al., 2011; Niu et al., 2017).

#### 8.3.1 Economic and commercialization barriers

##### 8.3.1.1 High development and production costs

1. Research and Development (R&D): Microbiome engineering requires significant investment in research, particularly for understanding microbial interactions, designing custom consortia, and testing their effectiveness in the field (Ke et al., 2021).
2. Challenge: High upfront costs may limit funding opportunities, especially in regions with limited access to research infrastructure (Compant et al., 2010).
3. Production Costs: Scaling up engineered microbial products for field application involves complex production processes (e.g., fermentation, strain optimization, and formulation), which can be cost-prohibitive for widespread commercial adoption (Compant et al., 2010).
4. Challenge: The high cost of production can make these products more expensive than traditional chemical inputs (e.g., synthetic fertilizers and pesticides), limiting farmer adoption, especially in developing countries (Ricroch et al., 2011).

#### 8.3.2 Market readiness and commercial viability

1. Supply Chain and Distribution: Commercialization of engineered microbial products requires a robust supply chain, including the development of effective formulation techniques to ensure microbial viability during storage and transport (Ricroch et al., 2011).
2. Challenge: Ensuring consistent quality and efficacy of microbial products across different geographical regions can be difficult, leading to product instability and variable field performance.
3. Economic Competition: The bio-based products market competes with cheaper, established alternatives like chemical fertilizers and pesticides. Even with growing interest in sustainability, market entry barriers remain high due to entrenched agricultural practices and low-price pressures from conventional products (Niu et al., 2017).

#### 8.3.3 Social and institutional barriers

##### 8.3.3.1 Farmer knowledge and adoption

1. Lack of Awareness and Understanding: Farmers may be unfamiliar with the benefits of microbiome engineering or may have limited knowledge of microbial products (Niu et al., 2017).
2. Challenge: Without proper training and education, there is resistance to adopting new agricultural technologies especially when traditional methods have been in practice for generations.
3. Perceived Risk and Trust: Farmers may perceive the use of engineered microbes as risky, especially in areas where traditional farming practices dominate, leading to reluctance in adopting new microbial technologies.
4. Challenge: Misunderstanding the long-term benefits and potential side effects of microbiome interventions can create distrust.

#### 8.3.4 Institutional support

1. Regulatory and Policy Frameworks: Developing clear, consistent regulatory guidelines for the approval of microbiome-based products is essential. However, the regulatory landscape for genetically engineered microorganisms or synthetic consortia is often underdeveloped or inconsistent across regions (Vorholt et al., 2017).
2. Challenge: Inconsistent or slow regulatory approval processes can delay product development and market entry, creating uncertainty for both producers and consumers.
3. Incentives and Subsidies: Government policies and subsidies play a significant role in facilitating the adoption of sustainable agricultural technologies. Without proper incentives, farmers may not find the financial justification for transitioning to microbiome-based solutions.
4. Challenge: Without clear institutional support or financial incentives, adoption of microbiome engineering technologies may be slow, especially in resource-constrained regions.

#### 8.3.5 Environmental concerns

##### 8.3.5.1 Impact on soil ecosystem health

1. Microbial Community Disruption: Introducing engineered microbial consortia may alter the natural balance of soil microbiomes, potentially reducing microbial diversity and affecting critical ecosystem functions such as nutrient cycling, pest suppression, and disease resistance (de Souza et al., 2016).
2. Challenge: Long-term impacts on soil health could include reduced resilience to environmental stressors (e.g., drought, pest outbreaks) and degradation of soil quality.

##### 8.3.5.2 Invasive species risk

1. Ecological Impact of Non-Native Strains: Engineered microbes, particularly those from outside the local environment, could become invasive, outcompeting native species or transferring novel genetic traits (e.g., antibiotic resistance) to other organisms (Herrera Paredes et al., 2018).
2. Challenge: If introduced microbes disrupt the ecosystem balance, there could be negative consequences on plant and animal health, making it difficult to reverse the impacts once they are established.

#### 8.3.6 Resistance and adaptation

1. Selection Pressure on Pathogens: Continuous use of microbial interventions that produce specific bioactive compounds (e.g., natural pesticides or antibiotics) could lead to the evolution of resistant strains of pathogens or pests (Toju et al., 2018).
2. Challenge: Over time, engineered microbes may lose efficacy, or pest/pathogen resistance could outpace the technology, reducing the benefits of microbiome engineering for intensification.

#### 8.3.7 Climate adaptability

1. Sensitivity to Climate Change: Engineered microbial consortia may perform well under controlled conditions but may fail under fluctuating climate conditions, such as extreme temperatures, drought, or flooding.
2. Challenge: The long-term climatic stability of engineered microbes must be thoroughly tested to ensure they can adapt to changing agricultural environments and still provide consistent benefits.

#### 8.3.8 Regulatory and ethical issues

##### 8.3.8.1 Regulation of genetically modified organisms

1. Stringent GMO Regulations: Many engineered microbial products fall under the category of genetically modified organisms (GMOs). Regulatory bodies in many countries have strict guidelines for the release of GMOs into the environment, requiring extensive safety assessments and environmental impact studies (Finkel et al., 2017).
2. Challenge: Navigating the complex regulatory landscape for GMOs, especially in regions where public perception of GMOs is negative, can slow the adoption of microbiome-based technologies.
3. Ethical Concerns: The use of genetically engineered microorganisms in agriculture raises ethical questions about the manipulation of natural organisms, the long-term safety of GMO microbes, and their potential impact on biodiversity.
4. Challenge: Public resistance to genetically engineered organisms may limit the broad adoption of these technologies, especially in markets where ethical concerns regarding GMOs are prominent.

#### 8.3.9 Long-term sustainability vs. immediate benefits

1. Short-Term Benefits vs. Long-Term Risks: While microbiome engineering can show quick results in terms of yield improvements and pest resistance, the long-term ecological stability of these interventions is less predictable (Zhang et al., 2019).

Challenge: Relying too heavily on engineered microbes might address immediate agricultural challenges but could result in unforeseen consequences for soil ecosystems, microbial diversity, or pest resistance in the long run.

## 9 Future scope and recommendations

A key novelty of this synthesis lies in linking synthetic microbiome design not only to crop stress tolerance and yield stability but also to broader climate mitigation outcomes including soil carbon sequestration and reduced reliance on chemical fertilizers and pesticides (Zhang et al., 2019). The review further distinguishes top-down enrichment and bottom-up rational design as complementary rather than competing strategies, outlining practical criteria for microbial selection, compatibility, and field persistence. By consolidating cropspecific case studies, emerging technologies, and translational challenges within a single conceptual framework, this work provides a roadmap for advancing synthetic microbiomes from experimental systems into field-ready solutions. Collectively, the insights presented position engineered rhizospheres as a central component of future climate smart and sustainable agricultural systems (Zhalnina et al., 2018; Wang et al., 2019; Ke et al., 2021).

Future studies should focus on using location-specific microbial compositions combined with adaptive SynCom design to make reliable field-scale applications dependent on experimental success (Wang et al., 2019). Long-term environmental monitoring will be of paramount importance in determining the ability and stability of operation for artificial microbiomes in response to changing soil and climate conditions. Omics data fusion with machine learning and artificial intelligence will not only forecast microbial interactions but will also optimize community composition and predict field results. CRISPR-based genetic transformation, chip-based laboratory systems, and microbioreactors have made it easier to seek out new plant-microbe collaborations (Banerjee et al., 2019). In addition, the establishment of artificial microbial communities together with precision agricultural technologies like IoT soil sensors and data-driven decision-making tools allows for real-time monitoring of rhizosphere health (French et al., 2021). There is a need for the harmonious coexistence of scientists, legislators, and farmers in drawing up regulations that form the means by which ethics and biosafety requirements will be met, ensuring that the high-tech methods augment not only the resilience of crops but protect and even enhance biodiversity and the balance of nature for times to come.

## 10 Conclusion

This review synthesizes recent progress made in synthetic microbiomes and bioengineered rhizospheres as transformative tools for climate-resilient agriculture. Complementing these single microbial inoculants and descriptive microbiome surveys, this work emphasizes a function-driven and ecology-aware framework where microbial communities are deliberately assembled, optimized, and stabilized to support plant performance under combined abiotic and biotic stresses. By focusing on core microbiome concepts, plant-microbe signaling networks, and ecological assembly principles, integrated with synthetic biology, multiomics, and data-driven design, this review underscores a shift from observational microbiome research to predictive and deployable microbiome engineering. Successful deployment of microbiome engineering requires coordinated efforts across the scientific, commercial, and policy spectrum, with careful attention to ecological safety, economic feasibility, and social acceptance. Stakeholders should act proactively and collaboratively to ensure that these technologies enhance crop productivity without compromising long-term environmental and societal sustainability.
